# A high-throughput nanoscale imaging platform for direct visualization and rapid measurement of telomeres at single-molecule resolution

**DOI:** 10.21203/rs.3.rs-11283393/v1

**Published:** 2026-10-28

**Authors:** Lilia Gheghiani, Andrey Mikheykin, Dylan Van Laeken, Daniel Guest, Kellie J. Archer, Harold C. Riethman, Jason Reed, Colleen Jackson-Cook

**Affiliations:** 1 Department of Pathology, Virginia Commonwealth University, Richmond, VA, USA; 2 Department of Physics, Virginia Commonwealth University, Richmond, VA, USA; 3 Evizia, Inc. Richmond VA; 4 Division of Biostatistics, College of Public Health, The Ohio State University, Columbus, OH; 5 School of Medical Diagnostic and Translational Sciences, Ellmer College of Health Sciences, Macon & Joan Brock Virginia Health Sciences at Old Dominion University, Norfolk, VA, USA; 6 Massey Comprehensive Cancer Center, Virginia Commonwealth University, Richmond, VA, USA; 7 VCU Institute for Engineering and Medicine, Virginia Commonwealth University, Richmond, VA, USA

**Keywords:** telomere measurement, telomere length, subtelomere mapping, nanoscale imaging, single-molecule analysis, chromosome-end profiling, biomarkers, atomic force microscopy

## Abstract

Telomere length is a biomarker of aging, cancer and telomeropathies, with some of the strongest health-related associations occurring at the extremes of the telomere-length spectrum. Critically short telomeres can drive chromosome instability, cellular stress and senescence, yet conventional assays largely provide population-average measurements that obscure these biologically important molecules, and the underlying heterogeneity of individual chromosome ends. In addition, amplification bias associated with highly repetitive telomeric sequences and inter-laboratory variability can limit measurement reproducibility. Although emerging technologies provide higher-resolution analyses, they often require high DNA input, specialized sample preparation, or complex instrumentation and bioinformatic tools, restricting broader adoption.

Here, we present a high-throughput single-molecule platform that overcomes these barriers through an automated, user-friendly nanoscale imaging workflow compatible with standard genomic DNA preparations. The method combines a unique dual CRISPR-Cas9 telomere boundary labeling strategy with amplification-free magnetic-bead-based enrichment, preserving the native telomere-length distribution while minimizing representation bias. Using only 500 ng of input DNA and 2–4 h of hands-on processing, the assay accurately resolves intact telomeres spanning ~50 bp to >50 kb within the same sample, enabling simultaneous characterization of the full telomere-length spectrum. It also demonstrates high reproducibility across technical replicates and independent operators (ICC up to 0.90). Validation in telomere-length models, replicative aging systems, and clinical biospecimens, together with subtelomeric barcoding for chromosome-arm assignment, establishes a scalable platform for chromosome-resolved telomere profiling, biomarker development, and future clinical implementation in aging, cancer, and telomere biology.

## Introduction

Telomeres are specialized nucleoprotein structures located at the ends of linear chromosomes, where tandem TTAGGG repeats and associated shelterin proteins form a protective chromosome-end cap. This cap prevents chromosome termini from being recognized as DNA breaks, thereby limiting degradation, end-to-end fusion, recombination, and inappropriate DNA damage signaling.^1–5^ Because telomeres shorten progressively with replication and are highly sensitive to oxidative and genotoxic stress, telomere length and integrity serve as dynamic determinants of genome stability, cellular senescence, aging, and disease susceptibility ^6–11^.

Adjacent to telomeres are subtelomeric regions, which are highly variable chromosome-end domains composed of repetitive elements, segmental duplications, telomere-variant repeats, and regulatory or transcribed sequences^12–18^ .These regions contribute to telomere maintenance and chromatin regulation through subtelomeric methylation, telomere-position effects, and TERRA-associated transcription, while their repeat-rich architecture also makes them recurrent sites of structural variation and instability^15,19–22^. Alterations affecting telomere length regulation are primary drivers of telomere biology disorders (TBDs), such as dyskeratosis congenita and idiopathic pulmonary fibrosis, as well as genomic instability in cancer and developmental abnormalities^9,23–28^.

Despite their clinical importance, accurate measurement of telomere length and subtelomeric architecture remains a significant challenge^29–33^. Conventional telomere-length assays, including Southern blot-based terminal restriction fragment (TRF) analysis and qPCR, provide bulk, average estimates rather than direct single-molecule measurements: TRF reports average telomere-length distributions across cell populations, whereas qPCR measures relative telomere repeat abundance as a telomere-to-single-copy gene ratio^29–31,34–36^. Although newer fluorescence- and sequencing-based approaches offer single-molecule, chromosome-arm-specific, or allele-resolved measurements, efficient analysis of intact, very long telomeres (>20 kb) from limited-abundance samples remain technically challenging, often requiring complex computational analysis (Table 1)^31–33,37–41^. To meet the needs for studies of telomere biology, a measurement platform must overcome several critical methodological hurdles:

### Sample Integrity and Input:

Efficient preparation of intact long chromosome ends is essential for applications in inherited disease genetics and oncology. Many methods, including long-read sequencing, require high DNA input and are highly impacted by fragmentation during preparation. There is a clear need for a method compatible with low-input (e.g., 500 ng) genomic DNA that preserves the structural integrity of the entirely intact telomeric regions.

### Telomere Size Range and Bias:

The ability to extend the range of measurable telomere sizes is required to reflect the actual distribution in a specimen. Non-amplified paths have considerable advantages over PCR-based methods, such as STELA and qPCR, which often fail to capture the full distribution, particularly very long telomeres, due to amplification bias. Moreover, it is important that the measurement is specific for the telomere, without including sub-telomeric regions (which confounds the interpretation of the data).

### Chromosome Identification:

Clinical outcomes are often driven by the shortest telomeres rather than the global average. Identifying which specific chromosome arms are losing protection allows for the detection of telomere-driven instability at its earliest stages. This requires the ability to interrogate very large molecules to combine telomere measurement with barcoding of adjacent subtelomeres, allowing identification of the chromosome of origin. Also, if a sentinel short telomere results in epigenetic changes of proximal non-telomeric loci, identifying the chromosome end could facilitate recognition of associated gene expression perturbations.

HS-AFM of barcoded chromosome-end fragments is a promising strategy to overcome these hurdles. AFM provides a complementary route to existing methods by directly visualizing DNA molecules at nanometer-scale resolution. However, implementing a quantitative molecular imaging method for this problem requires solving non-trivial issues regarding throughput and scanning dimensions.

In this work, we present an amplification-free HS-AFM workflow that enables direct, single-molecule profiling of chromosome ends. Our approach overcomes historical limitations through three key innovations:

### Targeted Enrichment and Surface Engineering:

By modifying protocols used in long-read sequencing to preserve large molecules and employing a spatially directed strategy using a photocleavable linker-magnetic bead system, we achieve efficient concentration of target molecules directly onto the imaging surface. We also developed an automated washing method that preferentially removes contaminants and linearizes surface-captured molecules for measurement.

### Automated Large-Area High-Speed Imaging:

Traditional fast AFM interrogates small areas rapidly but requires extensive stitching of image tiles to fit large DNA molecules. We developed a novel, large-area fast-scanning AFM that increases the linear dimensions of a single image tile by 24x and the area by 140x compared to previous implementations, maintaining nanometer registration without the slow process of computational tiling. The compact and fully automated imaging system supports walk-away analysis after sample loading.

### Direct Sequence Labeling:

We adapted a CRISPR-Cas9 DNA labeling strategy to target telomere-subtelomere junctions and incorporated nickase-based strand-displacement barcoding for chromosome-end identification. We demonstrate that this labeling scheme is robust and reproducible within our sample preparation workflow.

We validated this assay across multiple reference cell lines and whole-blood genomic DNA and demonstrated its ability to resolve biologically meaningful differences in telomere-length distributions, while creating a framework for chromosome-end-resolved telomere analysis.

Finally, our approach offers significant practical advantages (Table 1). It does not require an external standard to generate base-pair measurements or telomere ratio values, thereby eliminating a major source of lab-to-lab variability inherent in qPCR, TRF or flowFISH approaches. Furthermore, the workflow is relatively time- and cost-effective (estimated consumable costs are approximately $15–$20 per sample), achieving efficiency without the need for complex, computationally intensive bioinformatics pipelines or the high consumable costs associated with nanopore sequencing. Together, these results establish a direct, accessible method for chromosome-end-resolved analysis of telomere and subtelomere architecture.

## Results

### HS-AFM imaging workflow enables direct single-molecule measurement of intact telomeres

The HS-AFM workflow was designed to preserve the full spectrum of telomere lengths, minimize size-dependent bias, and enable precise end-to-end measurement of individual telomeres. To achieve this, we first evaluated DNA extraction methods for their ability to preserve long genomic DNA molecules and maintain representation of long telomeres during sample preparation (Supplementary Fig. 1 A&B). HS-AFM imaging showed that precipitation-based DNA extraction (Puregene, Qiagen) and high-molecular-weight DNA extraction (Monarch, NEB) preserved long DNA molecules, including fragments approaching megabase scale, whereas column-based extraction (QIAamp, Qiagen) reduced recovery of the longest genomic DNA molecules (Supplementary Fig. 1 A&B). Based on these results, both the Puregene and Monarch extraction methods were selected for subsequent analyses (Fig. 1, step 1). Extracted genomic DNA was subsequently digested to generate defined telomeric fragments containing adjacent subtelomeric sequence (Fig. 1, step 2). Site-specific restriction digestion was selected over mechanical fragmentation to avoid random DNA shearing that could disrupt the telomere–subtelomere junction. Several restriction enzymes, including those commonly used for terminal restriction fragment analysis, were evaluated (Supplementary Figs. 2–4). Among the enzymes tested, PacI produced a 20–50 kb terminal-fragment profile with sufficient adjacent subtelomeric sequence for downstream analysis (Fig. 1, step 2).

Telomeres were then selectively captured from bulk genomic DNA using a modified oligo-tagging strategy based on the previously described TeloTag approach^41^. A modified biotinylated oligonucleotide containing a telomere-complementary sequence and a photocleavable linker was ligated to the native single-stranded 3′ overhang, enabling selective capture on streptavidin magnetic beads and subsequent release of telomeric fragments for HS-AFM imaging (Fig. 1, step 3). Enrichment was quantified by qPCR as the relative abundance of telomeric DNA recovered in the bead-bound fraction compared with the unbound fraction (Supplementary Fig. 5 & 6B). Optimization of the genomic DNA-to-oligonucleotide ratio identified 1 μg genomic DNA and 33 nM oligonucleotide as optimal ratio conditions for enrichment (Supplementary Fig. 6A–B). Importantly, exonuclease I pretreatment, which removes the native 3′ telomeric overhang, abolished pull-down, demonstrating that capture depends on ligation to the native 3′ single-stranded terminus rather than to internal telomeric repeats or nonspecific genomic fragments (Supplementary Fig. 6C). Telomere PNA-FISH provided qualitative support for enrichment specificity, with telomere-positive fragments observed more frequently in the post-enrichment fraction than in bulk input DNA and pre-enrichment PacI-digested samples (Supplementary Fig. 6D).

For precise end-to-end measurement of individual telomeres, we established a unique CRISPR–Cas9 labeling strategy that defines both telomere boundaries (Fig. 1, step 4). Under cleavage-inactive conditions, Cas9 remains stably bound to target DNA, forming a large protein–DNA complex that is readily visualized by HS-AFM and can therefore be used as a programmable molecular marker^42^. Sequence analysis across the HG002 paternal, HG002 maternal, and CHM13 T2T reference assemblies identified a recurrent GC-rich18-bp motif at the telomere–subtelomere junction of most chromosome arms, which is part of a telomere-associated minisatellite repeat (29-mer) corresponding to a TERRA promoter element ^46–48^, providing a marker for the proximal telomere boundary (Supplementary Fig. 7A, B). To define the distal boundary, a 15-nt version of this motif was incorporated into the terminal oligonucleotide tag, creating a CRISPR-targetable sequence at the chromosome terminus (Supplementary Fig. 7D). Among the Cas9 complexes tested, HiFi Cas9 produced the most robust labeling at both boundary sites, with efficiencies exceeding 96% under optimized conditions (Supplementary Figs. 7 E, F, G and Supplementary Fig. 8).

To maximize image quality and telomere recovery while preserving DNA integrity, we optimized bead selection, removal of residual free oligonucleotides and unbound Cas9 complexes, and UV-release conditions. Among the cleanup methods evaluated, 0.22 μm dialysis most effectively removed residual free oligonucleotides and unbound Cas9 complexes while preserving intact DNA molecules (Supplementary Fig. 9A). Among five streptavidin magnetic bead types tested, M280 beads provided the best balance between efficient telomere capture and low imaging background (Supplementary Fig. 9B). Telomeric fragments were released from the beads by 365-nm UV cleavage of the photocleavable linker, enabling recovery of captured material under conditions expected to minimize DNA photodamage (Fig. 1, step 5). Control experiments showed that exposure to 365-nm UV for up to 30 min did not produce detectable DNA fragmentation, as assessed by HS-AFM and gel electrophoresis (Supplementary Fig. 10A). Sequential release experiments further demonstrated that 2 min of UV exposure was sufficient to recover the majority of bead-bound telomeric material (Supplementary Fig. 10B). Under these optimized conditions, 500 ng of genomic DNA yielded sufficient telomere recovery for HS-AFM analysis.

To improve imaging throughput and measurement efficiency, we implemented magnetically directed deposition to concentrate enriched telomeric fragments within the HS-AFM imaging field (Fig. 1, step 5). By increasing the local density of target molecules on the imaging surface, this approach reduced the scan area required for data collection and increased the number of analyzable molecules per image. Following UV release onto mica, controlled buffer flow straightened DNA molecules and facilitated contour tracing for accurate length measurements. This step was performed using an automated washing device to standardize directional buffer flow and improve protocol reproducability. Depositions of short, long, and mixed DNA populations confirmed that the workflow retained molecules across a broad size range without preferential loss of longer (or shorter) fragments (Supplementary Fig. 10C).

Together, these results establish an amplification-free platform for direct single-molecule measurement of telomeres by nanoscale imaging. By preserving long genomic DNA molecules, selectively enriching telomeres through their native 3′ overhang, defining distal and proximal telomere boundaries using dual CRISPR–Cas9 labeling, and enabling quantitative HS-AFM analysis from as little as 500 ng of genomic DNA, the workflow overcomes key limitations of bulk, amplification-based, and sequencing-based approaches.

### HS-AFM resolves telomere-length heterogeneity and passage-associated shortening in reference cell models

Having established the HS-AFM workflow, we applied it to a reference panel of human DNA samples spanning long-, intermediate-, and short-telomere states previously reported in the literature ^32,37,49–53^ (Supplementary table 1, Supplementary Fig. 11C, D). The panel included both lymphoblastoid and fibroblast-derived models, enabling assessment of telomere-length distributions across distinct cellular backgrounds. HS-AFM revealed broad single-molecule telomere-length distributions in every sample, indicating substantial heterogeneity rather than uniform telomere populations (Fig. 2A–C). The expected biological differences among models were readily apparent. The long-telomere models, 1301 and BJ, exhibited the largest distributions, with median telomere lengths of 5.8 kb and 4.9 kb, respectively. Approximately half of the measured telomeres exceeded 6 kb, and 22% were longer than 10 kb, whereas telomeres shorter than 500 bp were not detected (Fig. 2A–C). IMR-90 and HG002 displayed intermediate profiles, with median telomere lengths of approximately 4 kb and individual telomeres extending up to 25 kb, consistent with previous reports (Supplementary table 1 and Fig. 2A–C). In contrast, progeroid and dyskeratosis congenita (DC) samples showed a marked shift toward shorter telomeres despite their younger ages at collection, with median lengths of 1.7 kb and 1.3 kb, respectively, and ~30% of measured telomeres shorter than 1 kb (Fig. 2A–C). These findings demonstrate that HS-AFM captures both differences in median telomere length and the distributional features that distinguish long-, intermediate-, and telomere-dysfunction states.

Technical comparisons supported the robustness of the workflow and highlighted the importance of DNA integrity. Puregene and Monarch extraction methods produced comparable telomere-length distributions, whereas QIAamp extraction underrepresented longer telomeric fragments, consistent with reduced recovery of high-molecular-weight DNA (Supplementary Fig. 11A). Extraction replicates yielding different DNA recoveries generated comparable telomere-length distributions, indicating that variation in extraction yield did not substantially affect HS-AFM measurements (Supplementary Fig. 11B). Independent analyses performed by two technicians also produced broadly comparable telomere-length distributions and size-class assignments (Supplementary Fig. 11B). Reproducibility analysis based on the median telomere length of independent workflow repeats demonstrated high within-condition agreement, with an intraclass correlation coefficient (ICC) of 0.901. Residual repeat-to-repeat variation accounted for only 9.9% of the total variance, indicating strong consistency across independent workflow repeats performed using the same extraction and technician. Together, these findings support the high repeatability and robustness of HS-AFM telomere-length measurements across independent workflow repetitions.

We next assessed whether HS-AFM could detect telomere shortening during culture expansion. In IMR-90 fibroblasts, a well-established model of replicative telomere attrition, median telomere length decreased from 3.7 kb at passage 11 (p11) to 0.8 kb at passage 44 (p44), corresponding to an estimated loss of ~89 bp per passage (Fig. 2D, E). This value is consistent with a published estimate of 105 ± 17 bp per population doubling by mean TRF analysis in standard IMR-90 cultures^43^. Consistent with progressive shortening, the fraction of telomeres shorter than 1 kb increased from 3.2% to 59%, whereas the fraction longer than 6 kb decreased from 27% to 2.1%. A similar passage-associated redistribution was observed in HG002 cells. Median telomere length decreased from 3.9 kb at p4 to 1.3 kb at p16, corresponding to an estimated loss of ~219 bp per passage (Fig. 2D, E). This change was accompanied by an increase in telomeres shorter than 1 kb from 12% to 40% and a decrease in telomeres longer than 6 kb from 28% to 1.3%. The most pronounced shortening occurred in DC patient-derived cells (Fig. 2D, E). Median telomere length declined from 3.4 kb at p1 to 1.3 kb at p4, corresponding to an estimated loss of ~688 bp per passage. This rapid attrition is consistent with the defective telomere maintenance characteristic of dyskeratosis congenita^44^. The proportion of telomeres shorter than 1 kb increased from 14% to 40.7%, whereas the fraction longer than 6 kb decreased from 28% to 6%.

Together, these results demonstrate that HS-AFM resolves biologically distinct telomere-length states and detects dynamic telomere shortening during culture expansion. Across reference models, the abundance of very short telomeres emerged as a particularly sensitive indicator of telomere attrition, capturing distributional changes that were not fully reflected by median telomere length alone.

### HS-AFM workflow enables single-molecule telomere profiling from primary biospecimens

We next asked whether the HS-AFM workflow could be applied directly to primary blood-derived DNA, a key requirement for future cohort-based and clinically relevant telomere studies. Starting from only 500 μL of whole blood, the workflow yielded sufficient material for telomere enrichment, boundary labeling, deposition, and single-molecule HS-AFM measurement (Fig. 3).

Analysis of donors aged 31–90 years revealed broad, donor-specific telomere-length distributions and an overall shift toward shorter telomeres with age (Fig. 3A,B). Average telomere length decreased from 3.7 kb in the 30–35-year group to approximately 3.0 kb at 45 years, 2.4 kb at 56–60 years, and 2.3 kb in donors aged 80–90 years (Fig. 3A,B). This age-associated trend was accompanied by a progressive increase in very short telomeres. The fraction of telomeres shorter than 1 kb increased from 9% in the 30–35-year group to 13% at 45 years, 20% at 56–60 years, and more than 25% in the oldest donors. This pattern was most pronounced in the 90-year-old donor, in whom nearly one-third of measured telomeres were shorter than 1 kb (Fig. 3B). Concomitantly, longer telomere classes became progressively depleted. Telomeres longer than 6 kb represented 27% of molecules in donors aged 30–35 years, compared with 20% at 45 years and approximately 11–12% in donors older than 56 years (Fig. 3B). The fraction of telomeres exceeding 10 kb similarly decreased from an average of 9.3% in the youngest donors to 3.0% in donors aged 80–90 years. Notably, telomeres longer than 50 kb were detected in one 31-year-old donor but were absent from older individuals (Fig. 3B, C). These findings indicate that age-associated shortening in whole blood is characterized not only by reduced average telomere length, but also by marked remodeling of the underlying single-molecule distribution. A qPCR analysis of the same samples likewise revealed an age-dependent reduction in telomere-associated signal (Supplementary Fig. 11E), consistent with the differences detected using the HS-AFM workflow. Donor-level analysis further revealed substantial inter-individual variation that was not fully captured by average telomere length.

Together, these results demonstrate that single-molecule telomere profiling can be performed from as little as 500 μL of whole blood. The workflow captures age-associated redistribution of telomere lengths, including the accumulation of sub-kilobase telomeres and depletion of long and ultra-long telomeres, while revealing donor-specific patterns that would be obscured by bulk or median-based measurements alone.

### HS-AFM allows simultaneous single-molecule measurement of telomere length and chromosome-end identity

Telomere length varies substantially between chromosome arms, yet most existing methods measure telomeres as a pooled population. To enable chromosome-end-resolved analysis, we developed a subtelomeric barcoding strategy that encodes proximal subtelomeric sequence information on the same DNA molecule used for telomere measurement (Fig. 4A).

Candidate chromosome-end barcodes were identified by analyzing predicted nicking-site distributions across HG002 maternal, HG002 paternal, and CHM13 chromosome-end assemblies using multiple nicking endonucleases (Fig. 4B; Supplementary Fig. 12). Among the enzymes evaluated, Nt.BspQI generated the most informative and readily distinguishable barcode patterns while maintaining spacing compatible with HS-AFM resolution. To convert predicted Nt.BspQI sites into features detectable by HS-AFM, nicked DNA was subjected to strand-displacement synthesis to generate localized DNA-tail structures at defined genomic positions. Several strand-displacing polymerases were evaluated (Supplementary Fig. 13A), and Klenow Fragment produced the most robust and reproducible DNA-tail formation. Using a linearized pBR322 substrate containing a single Nt.BspQI site at a known position, we optimized strand-displacement conditions and confirmed that DNA-tail structures localized accurately to the predicted site. Labeling efficiency increased with extension time, reaching 95% after 5 min (Supplementary Fig. 12B). HS-AFM resolved strand-displacement products as punctate features consistent with ssDNA, flap-like structures consistent with dsDNA, or mixed structures at the target site (Fig. 4C). The dsDNA-containing structures may arise from secondary synthesis events, including template switching during strand-displacement synthesis. Because Nt.BspQI cleaves a defined strand relative to its non-palindromic recognition sequence, the orientation of each recognition site determines which genomic strand is nicked and the direction of polymerase extension from the nick-associated 3′-OH. Thus, predicted Nt.BspQI site position and orientation together provide positional and directional information that can be incorporated into subtelomeric barcode assignment (Fig. 4B,D).

Subtelomeric DNA-tail barcoding was subsequently combined with CRISPR–Cas9 telomere boundary labeling on HG002 chromosome-end molecules. Individual DNA molecules therefore contained both telomere boundary labels and subtelomeric barcode features, permitting telomere length and chromosome-end identity to be determined from the same molecule. Comparison of observed DNA-tail patterns with predicted Nt.BspQI barcode maps identified molecules corresponding to chromosome ends 18q and 14q (Fig. 4D), demonstrating that chromosome-end-specific subtelomeric signatures can be preserved and decoded on the same molecules used for direct telomere-length measurement.

Together, these results extend the HS-AFM workflow beyond telomere-length measurement by permitting simultaneous single-molecule determination of telomere length and chromosome-end identity, establishing a framework for chromosome-end-resolved studies of telomere heterogeneity, subtelomeric architecture, and genome instability.

## Discussion

In this study, we established an amplification-free HS-AFM workflow for direct single-molecule analysis of telomere-containing DNA molecules. The platform integrates end-preserving genomic DNA preparation, selective enrichment through the telomeric 3′overhang, CRISPR/Cas9-defined measurement boundaries, and subtelomeric barcode mapping. Together, these elements address a central limitation of widely used telomere assays: the inability to directly measure telomeric DNA while preserving chromosome-end context on the same molecule.

A key technical feature of the workflow is selective capture of chromosome-end fragments through ligation to the native telomeric 3′ overhang. Loss of enrichment after exonuclease I treatment provides strong evidence that pulldown specificity depends on the presence of the overhang rather than nonspecific bead binding. The workflow was further strengthened by optimization of AFM-compatible bead chemistry, oligonucleotide cleanup, and UV-based release, which together enabled recovery of telomere-enriched DNA with minimal background. These steps are important because molecule integrity, background suppression, and surface deposition directly affect the interpretability of single-molecule AFM measurements.

We present a targeted deposition strategy that optimizes the capture of long DNA fragments and macromolecular complexes for HS-AFM analysis. Unlike standard diffusion-governed methods, this technique concentrates analytes within a minimal scanning field, drastically reducing acquisition time. Integrating this protocol with Cas9 binding allows for the highly precise definition of genomic regions of genomic regions of interest in DNA conformations amenable to feature mapping. Future development of this strategy may enable a multi-parametric analysis of complex genetic and epigenetic landscapes such as length polymorphisms, epigenetic status, regulatory protein-binding events and non-canonical DNA structures providing a level of structural context that is currently unattainable through bulk biochemical or sequencing-based assays. Beyond nucleic acids and protein–DNA interactions, this targeted deposition strategy is broadly adaptable to diverse macromolecular assemblies. By functionalizing affinity agents—such as antibodies or aptamers—with photocleavable biotin tags, this approach expands the operational scope of HS-AFM from genomics to single-molecule proteomics and multi-omic structural biology.

While Scanning Probe Microscopy (SPM) and its most prominent variant, Atomic Force Microscopy (AFM), have been among the cornerstones of biomolecular imaging for decades, their application was historically constrained by low temporal resolution. Recent engineering advancements in High-Speed AFM (HS-AFM) have mitigated these scanning speed limitations, facilitating the transition of this technology into a viable tool for routine clinical diagnostics. The workflow detailed herein is inherently scalable, featuring a high capacity for end-to-end automation across all procedural phases, from initial sample preparation to high-resolution imaging and subsequent bioinformatic analysis. This modularity may facilitate the seamless integration of the platform into both high-throughput commercial environments and routine clinical diagnostic pipelines

The nickase-mediated labeling strategy described here builds on prior AFM-based approaches in which sequence-specific nicking restriction enzymes were used to introduce topographical labels onto DNA molecules, enabling molecular identification from intramolecular label spacing^45^. The current protocol utilizes sequence-specific enzymatic nicking followed by strand-displacement polymerization, wherein the displaced DNA serves as the molecular label. This streamlined “barcoding” approach enables high-resolution identification of individual DNA molecules and may facilitate haplotype phasing and may facilitate detection of that occur within the sub-telomeric region, including inversions, translocations, and large-scale insertions or deletions. By bridging the resolution gap between classical cytogenetics and high-throughput sequencing, this protocol could provide a robust physical scaffold for the accurate orientation and independent orthogonal validation of *de novo* genome assemblies.

The use of CRISPR/Cas9 labeling to define telomere boundaries extends AFM from a descriptive imaging tool to a measurement platform. HiFi Cas9 provided the most efficient labeling under the conditions tested, and optimization of reaction chemistry further increased labeling rates to near-saturating levels. This allowed telomere length to be scored between defined molecular landmarks rather than inferred indirectly from bulk signal. Importantly, the resulting measurements resolved expected differences among long-, intermediate-, and short-telomere reference models and captured passage-associated telomere attrition in both lymphoblastoid and fibroblast samples.

The ability to profile whole-blood genomic DNA expands the potential utility of the method beyond cultured cell systems. Current telomere assays used in clinical or translational studies often rely on relative or bulk-average readouts. In contrast, the present workflow provides single-molecule distributions, making it possible to assess shifts in telomere size classes, depletion of long telomeres, or enrichment of critically short molecules. Such distribution-level information may be valuable in settings where average telomere length alone is insufficient to describe biologically meaningful variation.

Long-read sequencing and HS-AFM provide complementary information. Long-read sequencing offers nucleotide-level and allele-resolved analysis, whereas HS-AFM directly measures the physical length of individual telomeric tracts between defined molecular labels and preserves adjacent subtelomeric context on the same molecule. HS-AFM therefore avoids sequence-based repeat-length inference, while long-read sequencing provides base pair level sequence information that HS-AFM does not.^32,33,40,41^

An additional strength of the platform is preservation of subtelomeric information. Identification of a recurrent telomere–subtelomere junction motif provided a target for boundary labeling, while BspQI-based nickase barcodes created a route toward chromosome-end assignment. The integrated proof-of-principle demonstrates that telomere length and subtelomeric barcode features can be measured on the same molecule. This is particularly relevant because chromosome-end-specific dynamics are increasingly recognized as important in telomere biology, yet they remain difficult to capture with conventional assays.

Overall, our findings establish HS-AFM as a practical framework for direct telomere/subtelomere analysis at single-molecule resolution. By combining selective enrichment, molecular boundary labeling, and subtelomeric mapping, this approach bridges nanoscale structural precision with translational relevance and provides a foundation for studying telomere biology in a chromosome-end-resolved manner.

## Methods

### Reference Cell Lines

The 1301 lymphoblastoid cell line was purchased from Sigma-Aldrich. HG002, AG03259, and GM01775 lymphoblastoid cell lines were obtained from the Coriell Institute. AG03259 and GM01775 correspond to Hutchinson–Gilford progeria syndrome and dyskeratosis congenita, respectively. BJ fibroblasts were obtained from ATCC, and IMR-90 fetal lung fibroblasts were obtained from the Coriell/NIA Aging Cell Culture Repository.

Lymphoblastoid cell lines were maintained in RPMI-1640 medium supplemented with 10% fetal bovine serum (FBS) and 1% penicillin–streptomycin at 37°C in a humidified atmosphere containing 5% CO_2_. Fibroblast cell lines were maintained in Eagle’s Minimum Essential Medium supplemented with 15% FBS and 1% penicillin–streptomycin under the same conditions. Cells were harvested at approximately 10 million cells by centrifugation at 1,200 rpm for 8 min, washed once with phosphate-buffered saline (PBS), and pelleted by a second centrifugation step.

### Blood Donors

A total of 14 individuals were included in this study, comprising four twin pairs and six unrelated singletons ranging in age from 31 to 90 years. Twin participants were recruited through the Mid-Atlantic Twin Registry. Following informed consent under Virginia Commonwealth University Institutional Review Board protocol (Twin ACT (Acquired Chromosomal changes over Time) Study, HM20030175), participants completed health history, dietary, and lifestyle questionnaires and provided peripheral blood samples. Blood collection was performed by a healthcare provider selected by each participant. Samples were shipped overnight to the Jackson-Cook Cytogenetics Laboratory for processing.

### Genomic DNA preparation

Genomic DNA from cultured cell pellets or peripheral blood was extracted using the Monarch^®^ HMW DNA Extraction Kit (New England Biolabs, T3050L), Puregene (Qiagen), or QIAamp (Qiagen) according to the manufacturers’ instructions. DNA was eluted in 300 μL elution buffer and quantified using the Qubit^™^ dsDNA High Sensitivity Assay Kit (Thermo Fisher Scientific, Q33230), yielding concentrations of approximately 100–300 ng μL^−1^.

### Restriction enzyme selection for telomere preservation

To identify restriction enzymes that preserve telomeric and subtelomeric DNA, 200-kb chromosome-end intervals were extracted from the T2T-CHM13 v1.1 reference genome and the HG002 maternal and paternal assemblies. Candidate enzymes were evaluated based on cutting frequency, CpG methylation sensitivity, and preservation of the telomere–subtelomere junction and adjacent subtelomeric sequence. Enzymes examined included PacI, BamHI, PvuI, BspWI, AsiSI, NdeI, EcoRI, HinfI, and RsaI.

Predicted digestion patterns were compared across all three assemblies to identify enzymes that generated large terminal fragments while retaining both telomeric and proximal subtelomeric DNA. Candidate enzymes were further evaluated experimentally by digestion of genomic DNA followed by electrophoretic analysis on 0.6% agarose gels in 1× TAE buffer. Fragment sizes were estimated using a high-molecular-weight DNA ladder.

PacI was selected because it consistently generated large terminal fragments (>50 kb) while preserving subtelomeric telomere-associated sequence and the telomere–subtelomere junction. For subsequent experiments, 0.5 μg genomic DNA was digested with 1 μL PacI (NEB, R0547S) in 1× NEBuffer 4 (BSA-free) in a final reaction volume of 20 μL and incubated at 37°C for 1 h. The resulting fragments were used for telomere adaptor ligation.

### Telomere adaptor ligation (TeloTagging)

Photo-cleavable biotinylated adaptor oligonucleotide and phosphorylated telomeric splint oligonucleotides representing all six possible telomeric 3′-overhang permutations were synthesized by Integrated DNA Technologies. All oligonucleotides were resuspended in nuclease-free water at 200 μM. For adaptor annealing, 6 μL photocleavable oligonucleotide and 1 μL of each splint oligonucleotide were mixed with 1.5 μL 10× HiFi Taq Ligase buffer and 6.5 μL nuclease-free water. The mixture was denatured at 99 °C for 1 min and gradually cooled to room temperature at a rate of 1°C min^−1^. Telomere tagging was performed in 50 μL reactions containing 0.5 μg PacI-digested genomic DNA, 16.5 nM annealed adaptor duplex, 5 μL 10× HiFi Taq DNA Ligase reaction buffer, and 1 μL HiFi Taq DNA Ligase (NEB, M0647S). Reactions were incubated at 65°C for 5 min, followed by 15 cycles of 65°C for 1 min and 45°C for 3 min to promote ligation of the adaptor to native telomeric 3′ overhangs. Ligated DNA was purified by overnight dialysis against distilled water using a 0.22 μm mixed cellulose ester membrane (MilliporeSigma, HAWP04700).

### Cas9-mediated telomere labeling

Single-guide RNAs targeting the conserved 15-nt telomere–subtelomere junction motif were obtained from Integrated DNA Technologies (IDT) and prepared according to the manufacturer’s instructions. For each reaction, Cas9 ribonucleoprotein complexes were assembled by combining 0.2 μL sgRNA (50 μM), 0.2 μL EnGen^®^ Spy Cas9-HF1 protein (NEB, E3322S; 20 μM), and 1 μL 10× Cas9 reaction buffer (20 mM HEPES, 100 mM NaCl, 0.1 mM EDTA, pH 6.5), with nuclease-free water added to a final volume of 10 μL. The mixture was incubated at room temperature for 10 min to allow ribonucleoprotein complex formation. Ligated genomic DNA was brought to 1× Cas9 reaction conditions by addition of 10× Cas9 reaction buffer. Subsequently, 10 μL of the preassembled Cas9 ribonucleoprotein complex was added, and reactions were incubated at 37°C for 1 h to permit binding of Cas9 to the conserved subtelomeric target sequence. DNA cleavage was prevented by the absence of Mg^2+^ from the reaction buffer, which permits target-specific Cas9 binding while preventing Mg^2+^-dependent nuclease activity, as previously described^42^. Optimization of the Cas9-binding conditions is presented in Supplementary Figs. 7 and 8. To stabilize protein–DNA complexes for HS-AFM imaging, glutaraldehyde was added to a final concentration of 1%, followed by incubation at room temperature for 20 min. Crosslinking reactions were quenched with Tris buffer (3 M, pH 7.6) to a final concentration of 750 mM. Excess unbound Cas9 ribonucleoprotein complexes were removed by overnight dialysis against 1× Cas9 reaction buffer using a 0.22 μm mixed cellulose ester membrane (MilliporeSigma, GSWP02500).

### Telomere enrichment by streptavidin pulldown

Biotinylated, Cas9-labeled genomic DNA was enriched using Dynabeads^™^ M280 Streptavidin magnetic beads (Thermo Fisher Scientific, 11205D). For each reaction, a 5 μL bead suspension was washed three times with wash/binding buffer (PBS, 0.01% Tween-20, 0.2 mM EDTA, and 400 mM NaCl) and resuspended in 50 μL of the same buffer. The bead suspension was combined with 50 μL Cas9-labeled DNA and incubated for 60 min at room temperature with gentle rotation (10 rpm) to capture biotinylated telomere molecules.

Following magnetic separation, the unbound fraction was removed. Bead-bound DNA was washed once with 500 μL wash/binding buffer and three times with 500 μL TE buffer to remove non-specifically bound DNA. Beads were subsequently resuspended in 10 μL TE buffer for downstream analyses.

### Telomere molecule imaging

Telomere sizing experiments utilized the Precyse system **(ref** www.eviziadx.com**)**, an automated high-speed atomic force microscopy (HS-AFM)-based platform for rapid sizing and visualization of DNA molecules. For each sample, 3 μL of streptavidin bead-bound telomeric DNA was deposited onto a Precyse 1 × 3-inch mica substrate. A magnet positioned beneath the substrate was used to concentrate bead-associated DNA molecules within a defined imaging region. Following localization, UV illumination was applied to cleave the photocleavable linker and release captured telomere molecules from the magnetic beads.

Subsequently, 3 μL of Precyse deposition buffer was added to facilitate adsorption of released DNA molecules to the mica surface and incubated for 5 min. To promote straightening of DNA molecules, 1 mL of Precyse deposition buffer was applied across the substrate in a single direction. Samples were then washed and dried by air jet in an automated washing station and heated at 102°C for 10 min before imaging.

For each sample, 28 images were acquired. Individual images covered a 24 × 48 μm field of view with a pixel resolution of 2 × 2 nm. Typically, more than 500 labeled telomere molecules were imaged within 60 min, and up to eight samples could be analyzed in a single run. Following image acquisition, data were exported as 16-bit TIFF files for downstream processing and telomere length analysis.

### qPCR-based assessment of telomere enrichment

To assess telomere enrichment following streptavidin pulldown, quantitative PCR (qPCR) was performed using primers targeting telomeric repeats and the single-copy albumin gene as a reference control. Samples analyzed included input genomic DNA, the flow-through (unbound) fraction, and the bead-bound (enriched) fraction.

Reactions were assembled in 20 μL volumes using SYBR^™^ Green Master Mix (Bio-Rad, 1725270) according to the manufacturer’s instructions. Each reaction contained 10% of the unbound or enriched DNA fraction, 10 μL 2× SYBR Green Master Mix, and 1 μL primer mixture. The primer mixture consisted of 16 μL telomere primer pair (20 μM each), 4 μL albumin primer pair (20 μM each), and 40 μL nuclease-free water. Reactions were performed in technical triplicate.

#### qPCR primer sequences:

##### Telomere

Forward: 5′-ACACTAAGGTTTGGGTTTGGGTTTGGGTTTGGGTTAGTGT-3′

Reverse: 5′-TGTTAGGTATCCCTATCCCTATCCCTATCCCTATCCCTAACA-3′

##### Albumin

Forward: 5′-CGGCGGCGGGCGGCGCGGGCTGGGCGGaaatgctgcacagaatccttg-3′

Reverse: 5′-GCCCGGCCCGCCGCGCCCGTCCCGCCGgaaaagcatggtcgcctgtt-3′

#### qPCR cycling conditions

Thermal cycling was performed as follows: 95°C for 5 min; two cycles of 94°C for 15 s and 49°C for 15 s; four cycles of 94°C for 15 s and 62°C for 15 s; 20 cycles of 83°C for 15 s, 60°C for 15 s, and 72°C for 15 s with signal acquisition; followed by 30 cycles of 94°C for 15 s, 83°C for 15 s, and 87°C for 15 s with signal acquisition. Melt-curve analysis was performed from 95°C with 0.5°C increments and a 5 s hold at each step.

Relative telomere enrichment was determined using the ΔΔCt method, with telomeric signal normalized to albumin and expressed as fold enrichment relative to either the input DNA or unbound fraction.

### Fluorescence in situ hybridization for telomere enrichment validation

To confirm telomere enrichment following streptavidin pulldown, fluorescence in situ hybridization (FISH) was performed using a FITC-labeled telomere-specific peptide nucleic acid (PNA) probe (DAKO, K5327). Four DNA conditions were analyzed: undigested genomic DNA, PacI-digested DNA, DNA prior to pulldown, and DNA following pulldown.

DNA samples were deposited onto poly-L-lysine-coated glass slides and air-dried. Samples were fixed in 3.7% formaldehyde for 2 min at room temperature, washed in Tris-buffered saline, pretreated for 10 min, and dehydrated through a graded ethanol series (70%, 90%, and 100%; 2 min each). Following co-denaturation at 80°C for 3 min, slides were hybridized overnight at 37°C with 10 μL FITC-labeled telomere PNA probe in a humidified dark chamber.

Post-hybridization washes were performed in DAKO rinse and wash solution at 65°C for 5 min with agitation, followed by ethanol dehydration. Slides were mounted with ProLong^™^ Gold Antifade Mountant containing DAPI (Thermo Fisher Scientific) and imaged by fluorescence microscopy.

### Identification of a repeated telomere–subtelomere junction motif

To identify conserved sequence features at the telomere–subtelomere junction, chromosome-end sequences from the Telomere-to-Telomere CHM13 v1.1 assembly were analyzed using RepFind. For each chromosome end, a 100-kb interval extending inward from the telomeric terminus was screened for repetitive sequence elements, including short tandem and interspersed repeats. RepFind output tables were manually curated and compared across chromosome ends.

A repeated 15-bp sequence motif was identified at 41 of 46 chromosome ends when up to two mismatches were permitted. This motif corresponded to a previously described TERRA promoter-associated 29 bp tandem repeat sequence located near the telomere–subtelomere junction. To assess similarity across genomes, the same motif was queried in the HG002 maternal and paternal assemblies, where it was detected at comparable subtelomeric positions. The 15-bp repeated sequence motif was subsequently used as a telomere-subtelomere junction landmark and target site for Cas9-mediated labeling of telomere-containing molecules.

### Subtelomeric Nt.BspQI strand-displacement barcoding

Predicted Nt.BspQI recognition sites were mapped within chromosome-end intervals from the HG002 maternal, HG002 paternal, and CHM13 assemblies to generate subtelomeric barcode maps. To generate HS-AFM-detectable structural features at these sites, DNA substrates were nicked with Nt.BspQI and subjected to polymerase-mediated strand-displacement synthesis.

Initial optimization experiments were performed using pUC19 plasmid DNA containing a single Nt.BspQI site. pUC19 was incubated with Nt.BspQI in NEBuffer r3.1 at 50°C for 1 h, followed by enzyme inactivation at 80°C for 20 min and dialysis against deionized water for 1 h using a 0.1 μm membrane. Nicked pUC19 molecules were subsequently incubated with Phi29 DNA polymerase, Bst-XT WarmStart DNA polymerase, or Klenow Fragment (exo−) in manufacturer-recommended reaction buffers containing dNTPs. Reactions were performed under enzyme-specific incubation conditions, heat-inactivated, dialyzed against deionized water for 1 h using a 0.1 μm membrane, and analyzed by HS-AFM.

Strand-displacement conditions were further optimized using pBR322 DNA containing a single Nt.BspQI site at a defined position. pBR322 was linearized with BamHI, purified using AMPure beads, nicked with Nt.BspQI, and incubated with Klenow Fragment (exo−) at 37°C for 20 sec, 1 min, or 5 min. Reactions were heat-inactivated, dialyzed against deionized water for 1 h using a 0.1 μm membrane, and analyzed by HS-AFM.

For chromosome-end barcoding experiments, telomere-adaptor-ligated HG002 genomic DNA was nicked with Nt.BspQI and subjected to strand-displacement synthesis using Klenow Fragment (exo−). Following strand-displacement labeling, molecules were processed by Cas9-mediated labeling, streptavidin enrichment, and HS-AFM imaging as described above. DNA-tail positions detected by HS-AFM were compared with predicted Nt.BspQI site maps derived from the HG002 maternal, HG002 paternal, and CHM13 assemblies.

### Intraclass correlation coefficient

Using the HG002 cell line data, we fit a linear mixed-effects model for median telomere length, including technician and unique extraction as random effects. We obtained the variance components for technician, extraction, and residual error from the fitted model. Finally, we used these components to calculate the intraclass correlation coefficient (ICC). This metric quantifies both the correlation between replicates from the same extraction and technician, and the proportion of total variance attributable to the extraction process.

## Supplementary Material

1

## Figures and Tables

**Figure 1.**
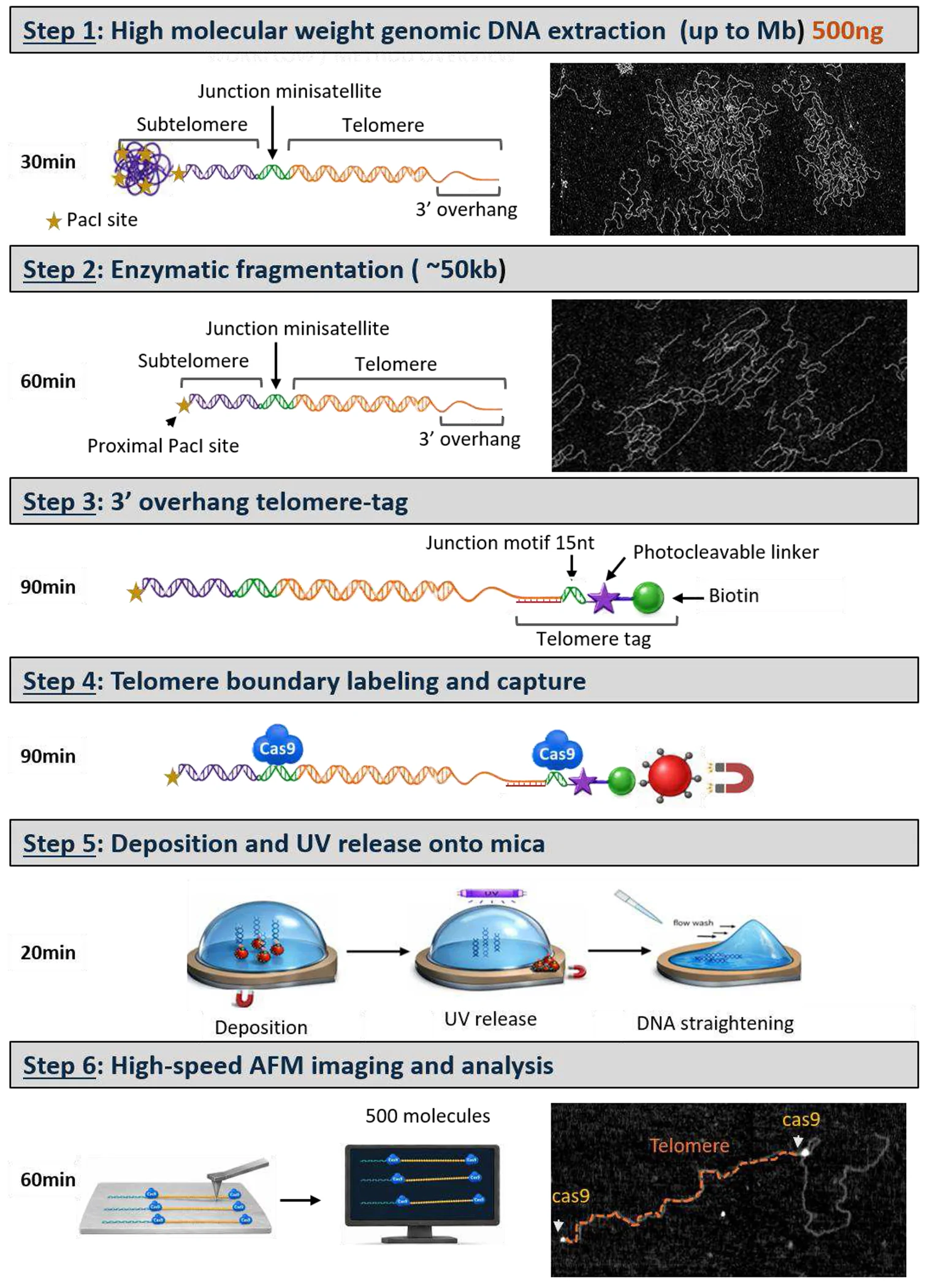
HS-AFM imaging workflow enables direct single-molecule measurement of intact telomeres. High-molecular-weight genomic DNA (500 ng) is enzymatically fragmented to generate chromosome-end molecules containing telomeric and subtelomeric regions. A telomere-specific tag is ligated to the native 3′ overhang, followed by telomere boundary labeling and capture using sequence-specific Cas9 markers and biotin-streptavidin enrichment. Captured molecules are deposited onto mica, released by UV cleavage, straightened by buffer flow, and imaged by high-speed atomic force microscopy. This workflow enables direct visualization and measurement of individual telomere molecules.

**Figure. 2.**
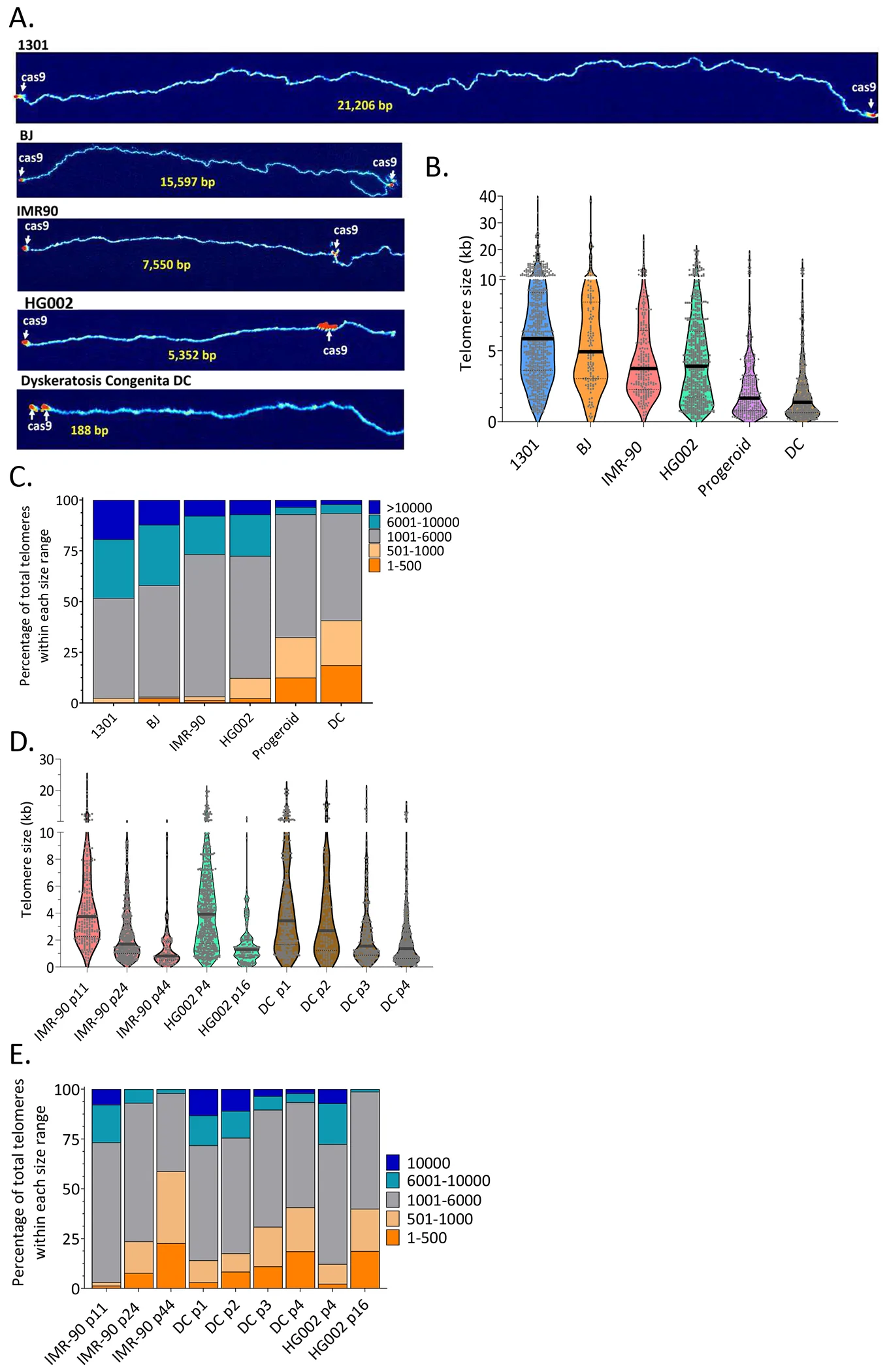
HS-AFM telomere profiling captures broad length variation with passage-associated shortening. (A) Representative HS-AFM images of individual telomeres from 1301, BJ, IMR-90, HG002, and dyskeratosis congenita (DC) cells. Cas9 labels mark the proximal and distal telomere boundaries, and measured telomere lengths are indicated. HS-AFM resolved individual telomeres in these images span more than two orders of magnitude, from 188 bp to >21 kb. (B) Single-molecule telomere-length distributions across reference and health condition-associated cell models. (C) Percentage of total telomeres within defined length categories for each cell model. (D) Single-molecule telomere-length distributions across different passage numbers in IMR-90, HG002, and DC cells. (E) Fraction of telomeres within defined length categories for the samples shown in D. For the distribution analyses shown in B and D, each point represents an individual telomere measurement; 400-500 telomeres were analyzed per sample.

**Figure 3.**
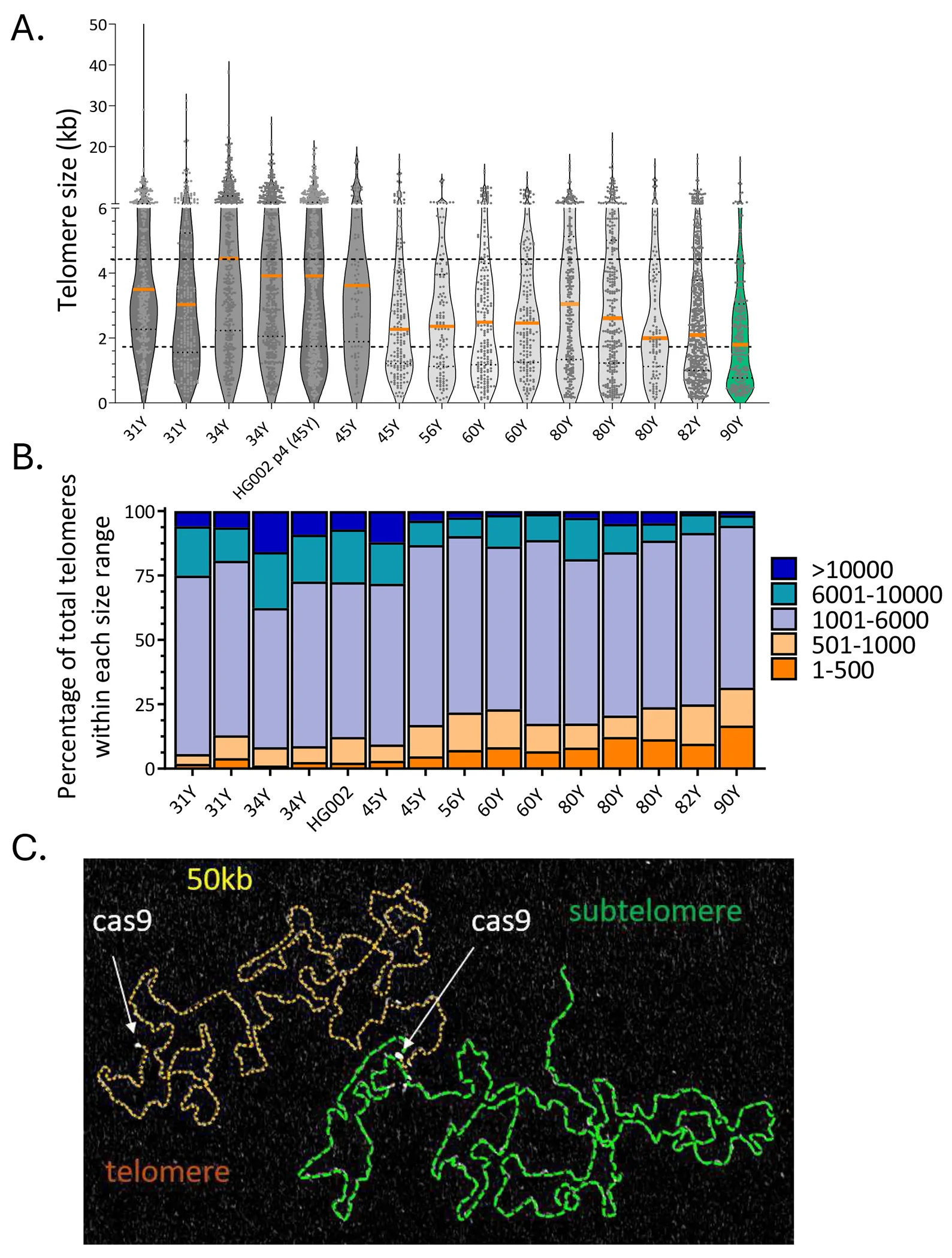
HS-AFM enables single-molecule telomere profiling from primary blood-derived biospecimens. (A) Single-molecule telomere-length distributions measured by HS-AFM in peripheral blood DNA obtained from donors aged 31–90 years. Each point represents an individual telomere measurement; 350-500 telomeres were analyzed per donor. Orange lines indicate median telomere length for each donor. The upper and lower dashed horizontal lines indicate the highest and lowest median telomere lengths observed across the donors, respectively. (B) Percentage of total telomeres within defined length categories for each donor. Age-associated telomere shortening is accompanied by accumulation of short telomeres and depletion of long and very long telomere classes. (C) Representative HS-AFM image of an ultra-long telomere (>50 kb) identified in a 31-year-old donor. Cas9 labels mark the telomere boundaries, enabling direct measurement of the telomeric tract (orange) relative to the adjacent subtelomeric region (green).

**Figure 4.**
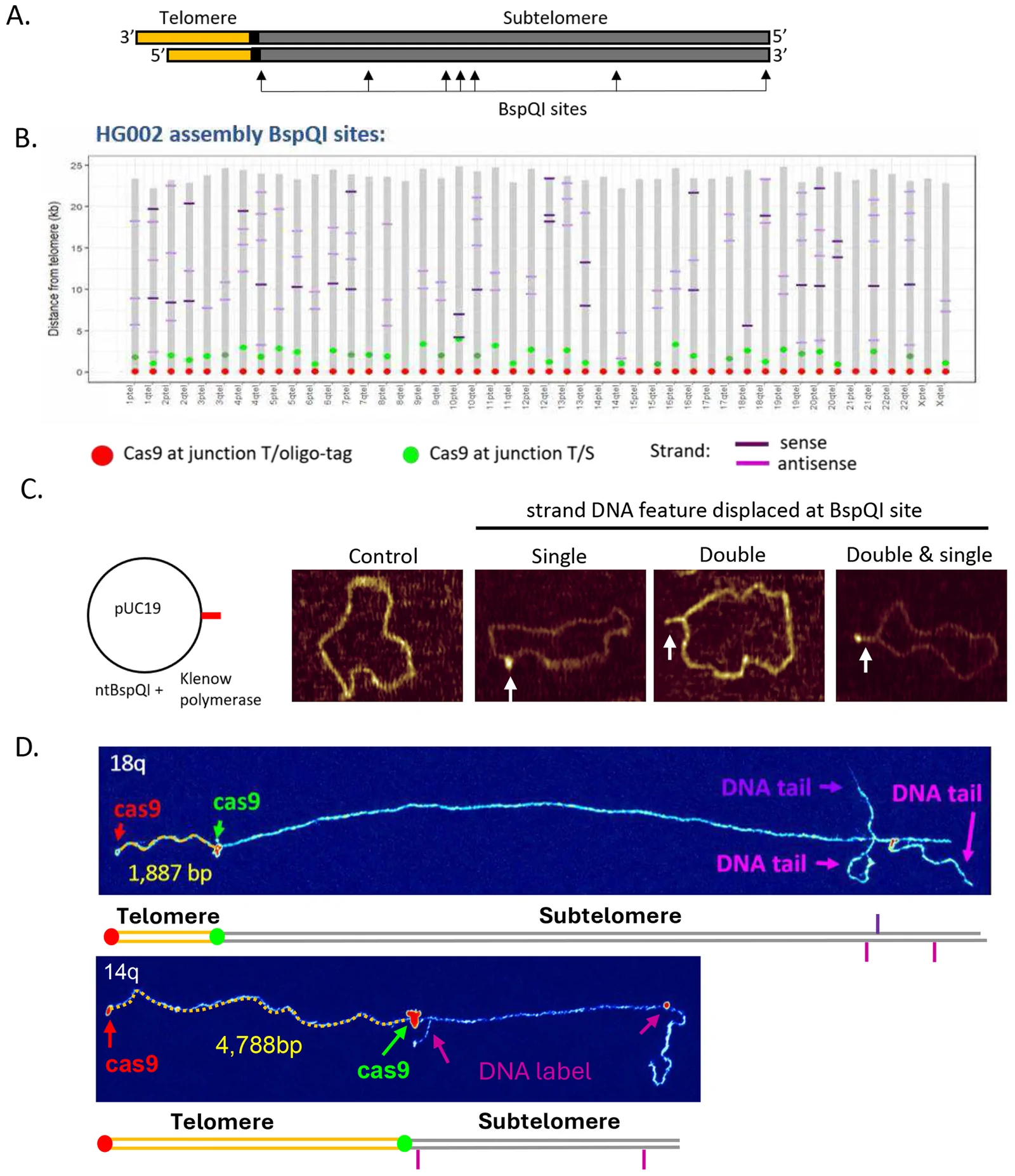
HS-AFM permits simultaneous single-molecule measurement of telomere length and chromosome-end identity. (A) Schematic of the subtelomeric barcoding strategy. Predicted Nt.BspQI sites within proximal subtelomeric regions are converted into DNA-tail barcode features by nicking and strand-displacement synthesis, enabling chromosome-end identification on the same molecule used for telomere-length measurement. (B) Predicted Nt.BspQI barcode architecture across HG002 chromosome ends. The positions and strand orientation of Nt.BspQI sites relative to the telomere boundary generate chromosome-end-specific barcode signatures compatible with HS-AFM resolution. Red and green circles indicate the distal and proximal Cas9 boundary labels, respectively. Purple bars denote Nt.BspQI nicking sites on the sense (dark purple) or antisense (lighter pink/purple) strand. (C) Conversion of Nt.BspQI sites into HS-AFM-detectable molecular features. Following Nt.BspQI nicking and Klenow-mediated strand-displacement synthesis, localized DNA-tail structures were generated at predicted positions. Representative HS-AFM images show punctate single-stranded DNA labels, double-stranded flap labels, and mixed strand-displacement products. Arrows indicate DNA-tail structures generated at the Nt.BspQI site. (D) Representative HG002 chromosome-end molecules combining subtelomeric barcoding with CRISPR–Cas9 telomere boundary labeling. Comparison of observed DNA-tail patterns with predicted Nt.BspQI barcode maps identified molecules consistent with chromosome ends 18q and 14q. Cas9 labels define telomere boundaries, DNA-tail features provide subtelomeric barcode information, and telomere lengths are indicated for each molecule.

**Table 1.** HS-AFM addresses key limitations of conventional telomere length measurement methods. Q-FISH, Flow-FISH, qPCR, TRF, long-read sequencing, and HS-AFM were compared based on measurement type, resolution, ability to provide subtelomeric information, detection of the shortest telomeres, sample input, and estimated cost per sample. Compared with existing approaches, HS-AFM uniquely combines amplification-free single-molecule telomere measurement with chromosome-end specificity, preservation of proximal subtelomeric context, and broad dynamic range. The reported cost per sample represents consumable costs only and does not include instrumentation, infrastructure, personnel, data-analysis, or reference-laboratory overhead.

Method	Primary Output	Sample Requirement	Sample Input Amount	Resolution	Subtelomere Information	Critically Shortest Telomere Detection	Consumable cost per sample (USD)
**Q-FISH**	Signal intensity per individual chromosome end	Intact metaphase cells	0.5mL blood	~1 kb *(single molecule)*	Limited *(chromosome-arm mapping via karyotyping)*	Limited *(signal-to-noise threshold)*	$50-150
**Flow-Fish** *(Clinical Diagnostic “Gold Standard”)*	Average telomere length per cell across a population	Intact single-cell suspensions	1 million cells	~1 kb *(bulk average)*	No	No *(measures cell-population mean)*	$50-100
**qPCR**	Relative telomere-to-single-copy gene* (T/S) ratio	Genomic DNA	50ng DNA	Bulk relative metric *(no bp resolution)*	No	No *(measures bulk molecule ensemble mean)*	$2-5
**TRF** (Historical Reference Benchmark)	Density distribution of telomeric restriction fragment lengths	High-molecular-weight genomic DNA	10ug DNA	0.5–1 kb *(bulk DNA ensemble)*	*Portion included in measurment (adds non-telomeric offset)*	No *(short fragments lost in gel background noise)*	$20-50
**Long-read sequencing**	Direct single-molecule sequence readout	High-molecular-weight genomic DNA	10-40ug DNA	Single base pair	Full sequence mapping and variant repeat profiling	Yes *(exact single-molecule readout down to 0 bp)*	$80-140
**HS-AFM**	Single-molecule DNA contour length	High-molecular-weight genomic DNA	500ng DNA	~3 bp/pixel *(single-molecule spatial)*	Physical contour and probe-based mapping	Yes *(direct single-molecule nanometer-scale imaging)*	$15-20

* Standard (unique copy gene) varies among labs

## Data Availability

All data supporting the findings of this study are available within the Article and Supplementary Information.

## Highlights

- Amplification-free HS-AFM workflow for single-molecule telomere analysis
- Low-input genomic DNA requirement
- Direct base-pair-scale measurement of telomere length
- Resolution of short-to-long telomeres across a broad range
- Capture of single-molecule telomere-length heterogeneity
- Integration of telomere length with chromosome-end identity
