# Supplementary material for: A high-throughput nanoscale imaging platform for direct visualization and rapid measurement of telomeres at single-molecule resolution
Source: Res Sq. 2026 Oct 28:rs.3.rs-11283393. Preprint. [Version 1] doi: 10.21203/rs.3.rs-11283393/v1 (PMC13734252; doi:10.21203/rs.3.rs-11283393/v1)
Supplement: Supplementary file 1 [file NIHPPrs11283393v1-supplement-1.pdf]

**Supplementary Table 1:** Average telomere length measurement for reference cell lines

| Cell line | TRF | Flow-FISH | TeSLA/ STELA | Optical mapping | Long-read sequencing | HS-AFM | Measurement references |
|---|---|---|---|---|---|---|---|
| **1301** | ~80 kb | ~25 kb | – | – | – | mean 6.9 kb (range: 0.5-40kb) passage 4 | TRF/Flow-FISH: [49]<br>HS-AFM: This study |
| **IMR90*** | 8.5 kb (early passage)<br>5.7 kb (late passage) | – | Chr17: ~1.52 kb | 7.2 kb (passage 17)<br>4.5 kb (passage 45)<br>4 kb (senescent) | IMR90 E6E7:<br>4.2 kb (passage 66)<br>2.7 kb (passage 106) | mean 4.7kb (range 0.2-23kb) passage 11<br><br>mean 2.3 kb (range 0.16-9.3kb) passage 24 | TRF: [50]<br>Chr17/STELA: [51]<br>Optical mapping: [37]<br>Long-read: [32]<br>HS-AFM: This study |
| **HG002** | 4–6 kb | – | – | – | ~4–5 kb | mean 4.5 kb (range 0.1-20kb) passage 4<br><br>mean 1.69 kb (range 0.079-11kb) passage 16 | TRF/long-read: [32]<br>HS-AFM: This study |
| **BJ** | 6.4 kb (early passage)<br>4.9 kb (senescent) | – | 4.7 kb | – | – | mean 6.3 kb (range 0.25-38kb) passage 4 | TRF: [52]<br>TeSLA: [53]<br>HS-AFM: This study |

** Note: The long-read sequencing values were measured in IMR90 E6E7 cells, an E6/E7-immortalized derivative that is distinct from the parental IMR90 cells used for the TRF, chromosome 17 STELA, optical-mapping, and HS-AFM measurements.*

*Abbreviations: TRF, terminal restriction fragment ( include a portion of subtelomeric region); Flow-FISH, flow cytometry–fluorescence in situ hybridization; TeSLA, telomere shortest length assay; STELA, single telomere length analysis; HS-AFM, high-speed atomic force microscopy.*

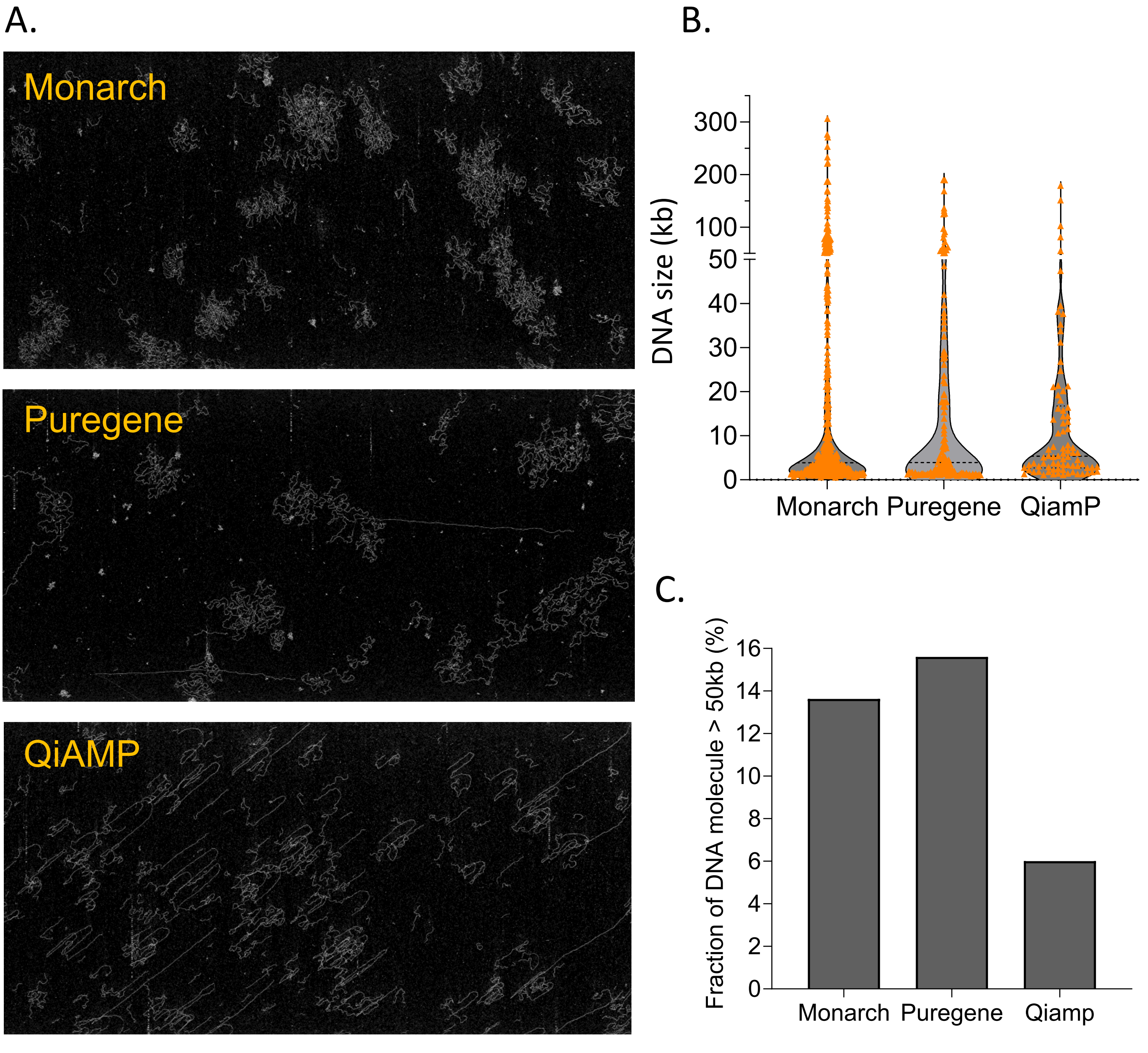


**Supplementary Fig 1. Monarch and Puregene extraction methods yield greater recovery of long genomic DNA molecules than QIAamp.** (A) Representative HS-AFM images of genomic DNA extracted using the Monarch, Puregene, and QIAamp methods. (B) Length distributions of individual genomic DNA molecules measured by HS-AFM following extraction with each method. Violin plots show the distribution of molecule lengths, with Monarch and Puregene preserving long DNA molecules, including fragments exceeding 50 kb, whereas QIAamp showed reduced recovery of the longest molecules. (C) Percentage of DNA molecules >50 kb recovered using each extraction method, demonstrating greater retention of long genomic DNA molecules by Monarch and Puregene compared with QIAamp

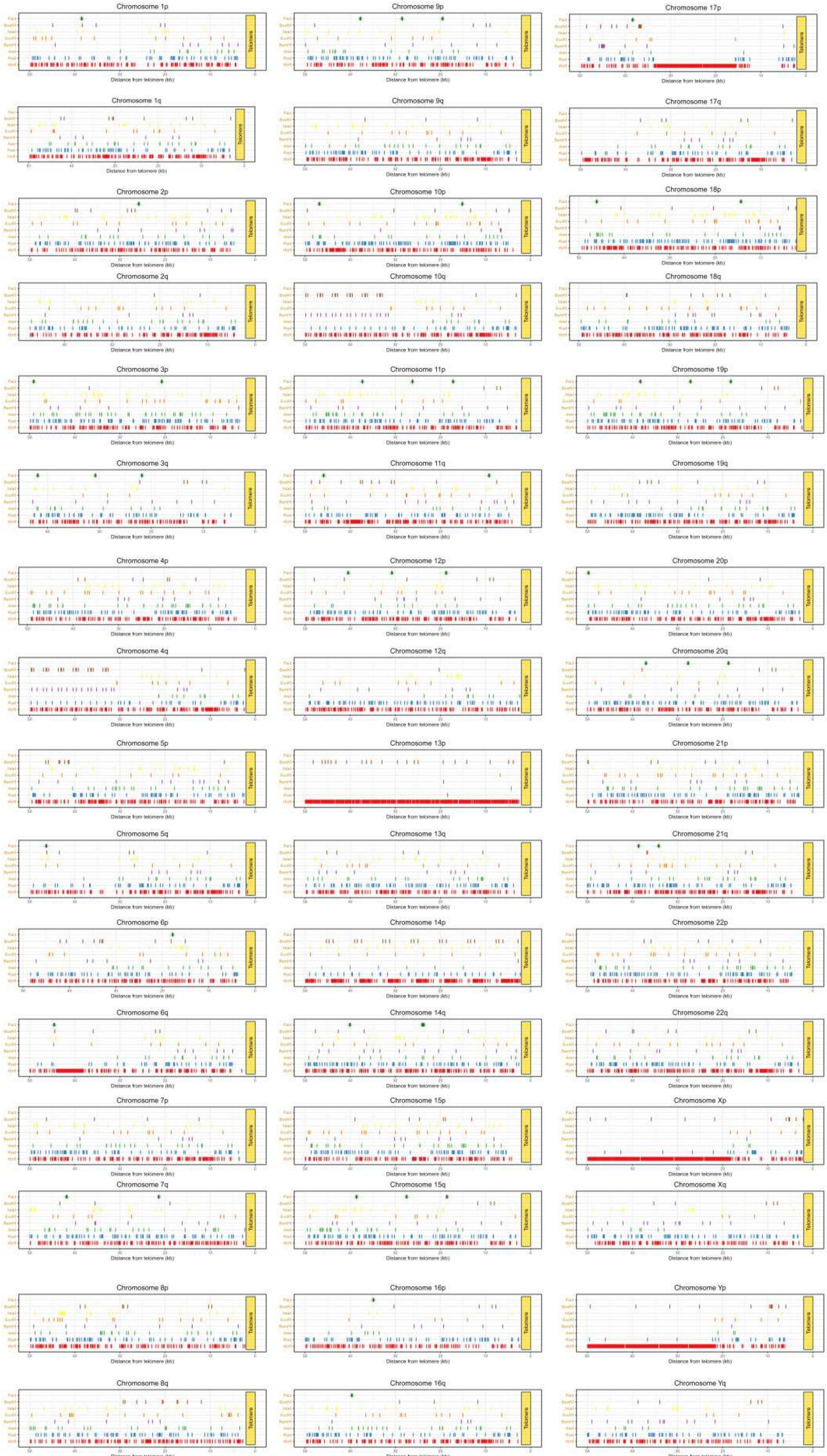


**Supplementary Fig 2. CHM13 chromosome-end restriction-site maps.** Genome-wide *in silico* restriction-site maps of CHM13 chromosome ends used to evaluate enzymes for end-preserving fragmentation. Analysis of predicted terminal-fragment profiles identified PacI as the optimal enzyme for generating 10–50 kb telomere-containing terminal fragments while retaining sufficient adjacent subtelomeric sequence for downstream analysis.

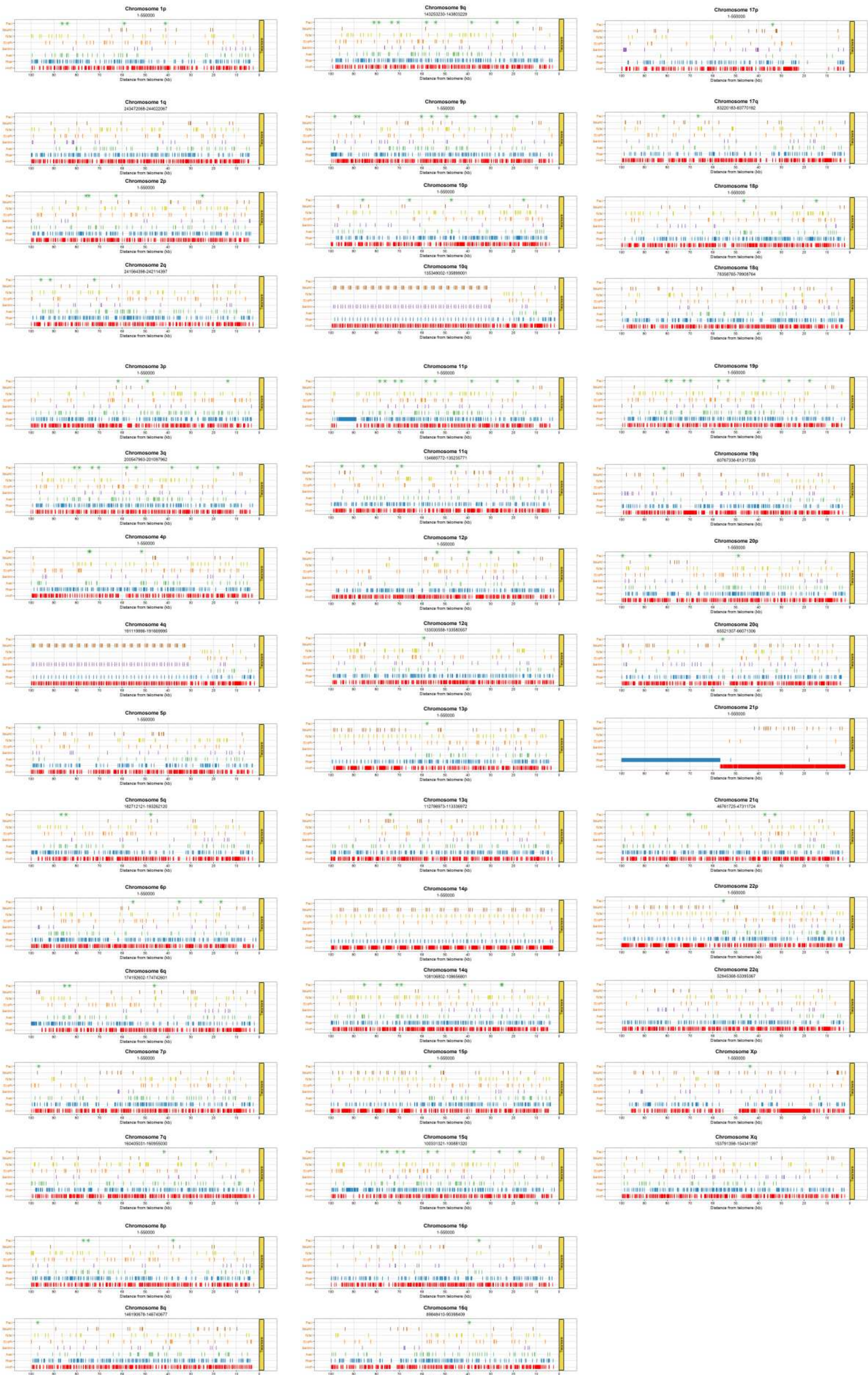


**Supplementary Fig 3. HG002 maternal chromosome-end restriction-site maps.** Genome-wide in silico restriction-site maps of HG002 maternal chromosome ends used to evaluate candidate restriction enzymes for generation of telomeric terminal fragments while preserving proximal subtelomeric sequence

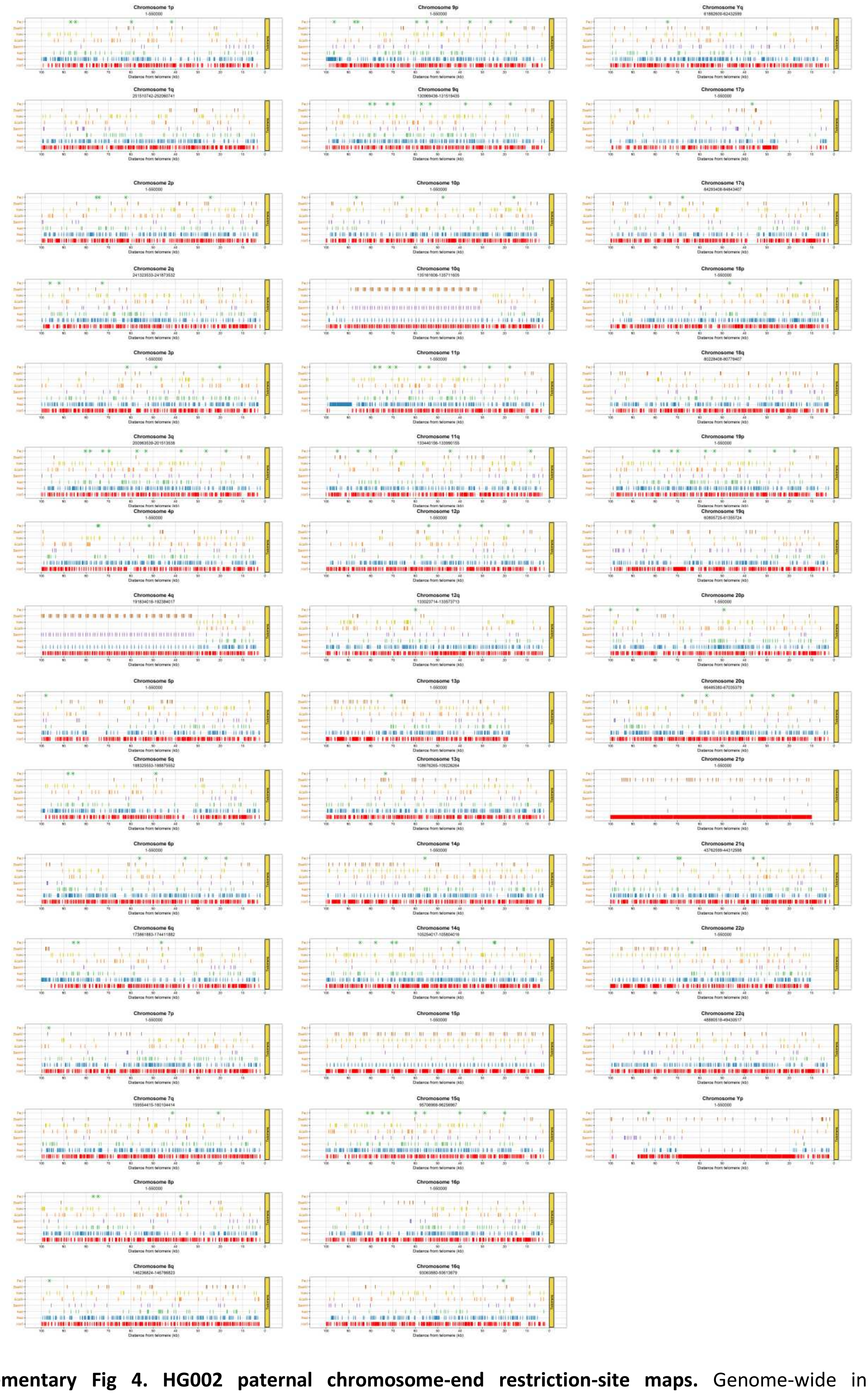


**Supplementary Fig 4. HG002 paternal chromosome-end restriction-site maps.** Genome-wide in silico restriction-site maps across HG002 paternal chromosome ends used to evaluate candidate restriction enzymes for generation of telomeric terminal fragments while preserving proximal subtelomeric sequence

A.

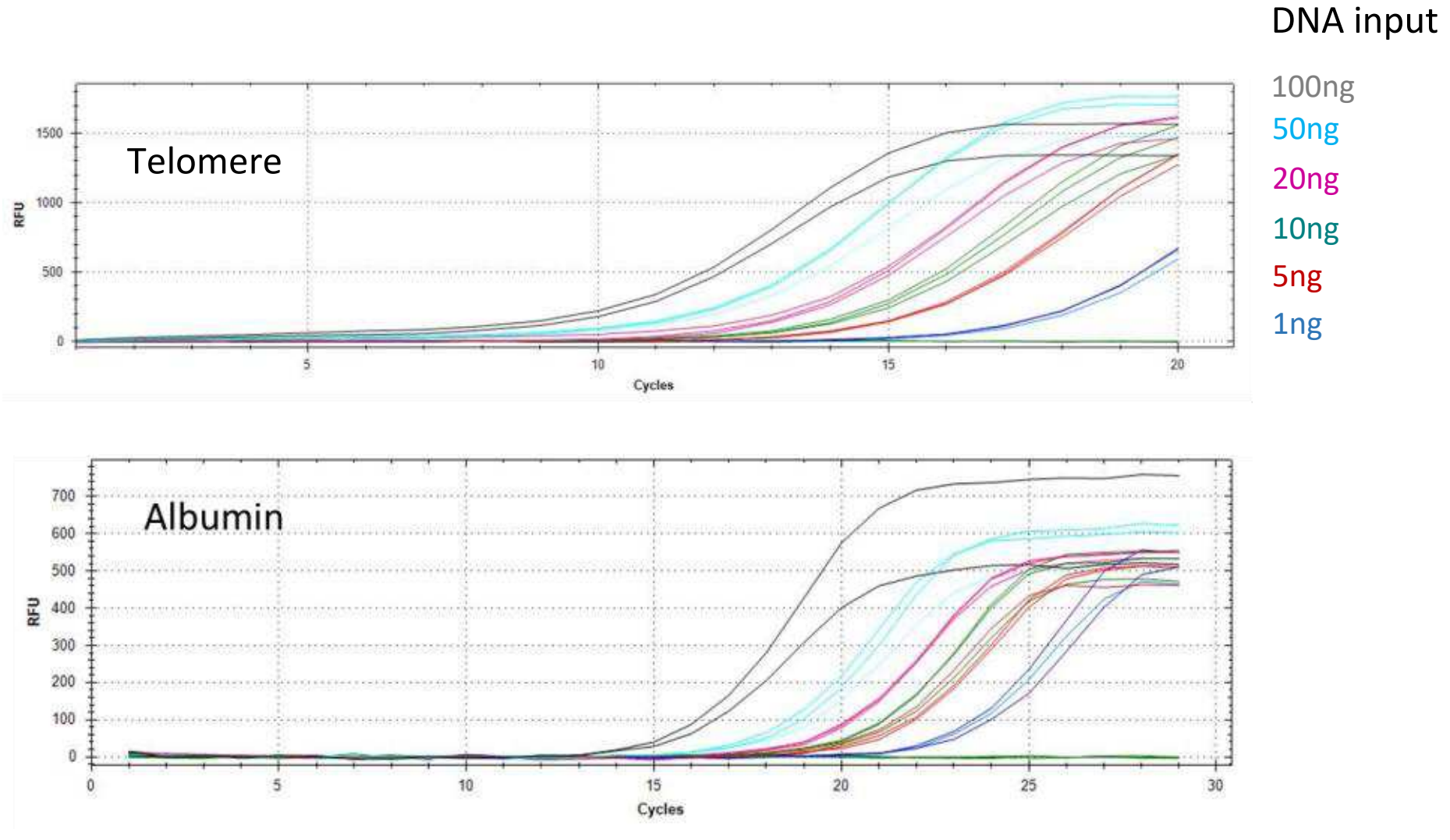


B.

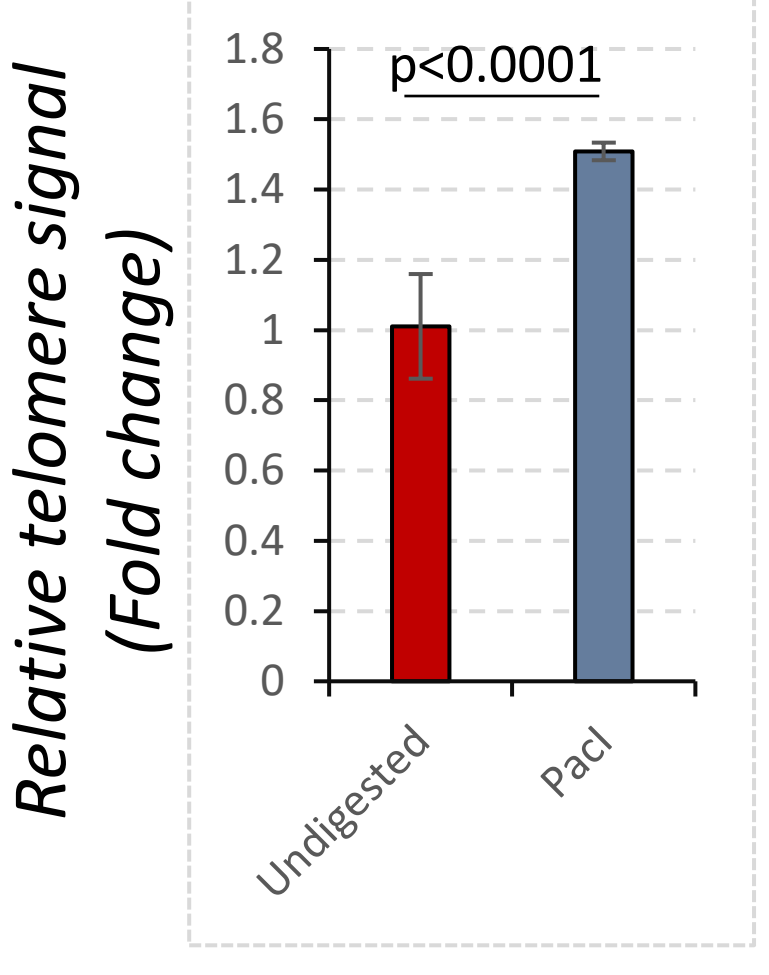


**Supplementary Fig. 5. Assessment of qPCR performance and the effect of PacI digestion.** (A) Representative telomere and albumin qPCR amplification curves across increasing DNA inputs (1–100 ng). (B) Relative telomere signal in undigested and PacI-digested genomic DNA, expressed as fold change relative to the undigested condition ($P < 0.0001$, two-tailed paired Student's t-test). Data are mean ± s.d. from three biological replicates analyzed in technical triplicate.

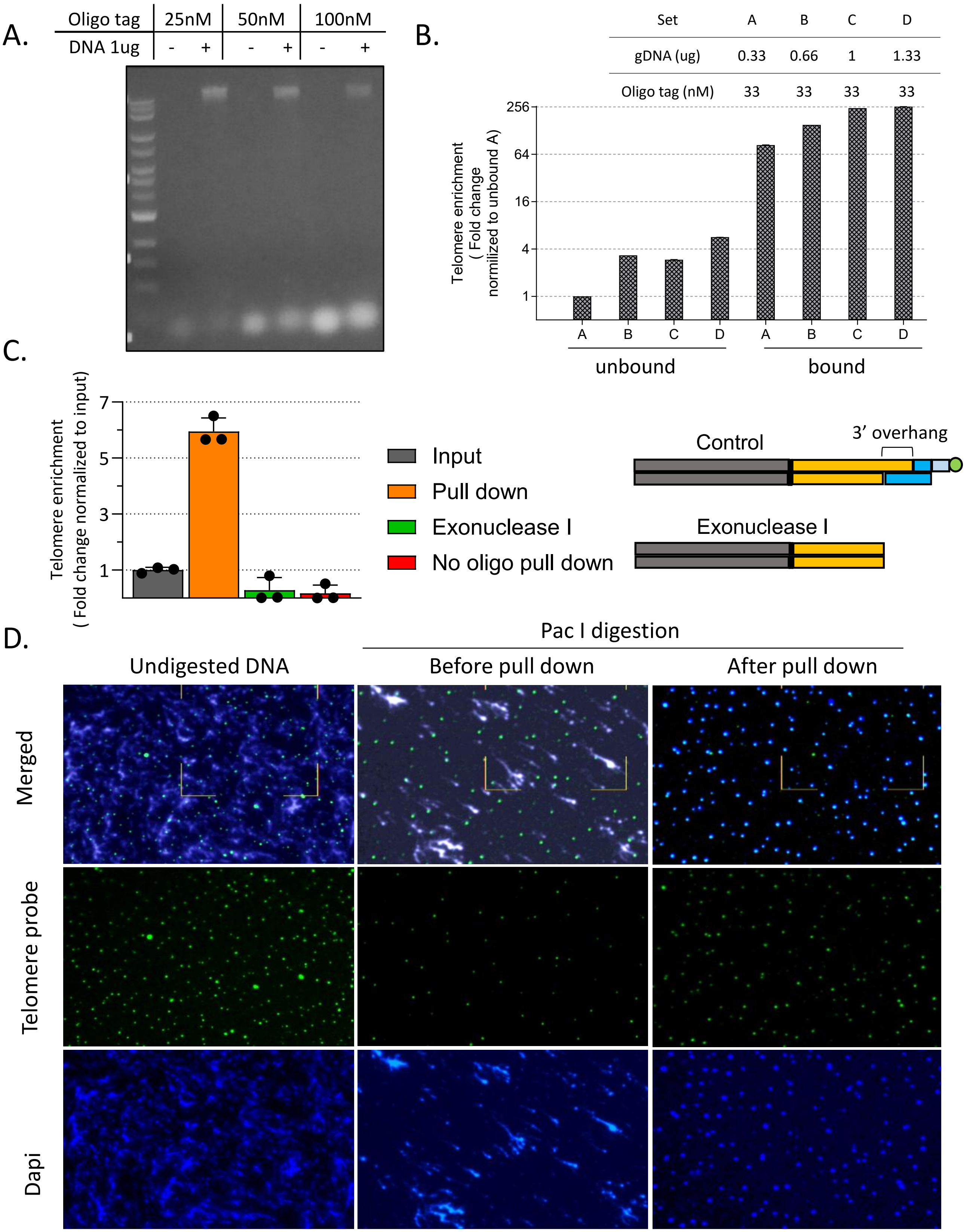


**Supplementary Fig 6. Specific capture of telomeric fragments** (A) Gel-based assessment of ligation of the biotinylated telomere-tag oligonucleotide to genomic DNA using increasing oligonucleotide concentrations (25, 50, and 100 nM) in the presence or absence of genomic DNA. (B) qPCR analysis of telomere enrichment in bound and unbound fractions across different genomic DNA and oligonucleotide input conditions, identifying conditions that maximize telomere capture efficiency. (C) qPCR analysis of telomere enrichment in input, pulldown, exonuclease I-treated, and no-oligo control samples. Removal of the native 3′ telomeric overhang by exonuclease I abolished enrichment, demonstrating that capture depends on ligation to the native chromosome-end overhang. A schematic of the exonuclease I treatment strategy is shown. (D) Representative telomere PNA–FISH images of undigested genomic DNA, PacI-digested DNA before pulldown, and the enriched fraction after pulldown. Telomere signals were detected predominantly in the enriched fraction, consistent with the specificity of telomere capture.

A.

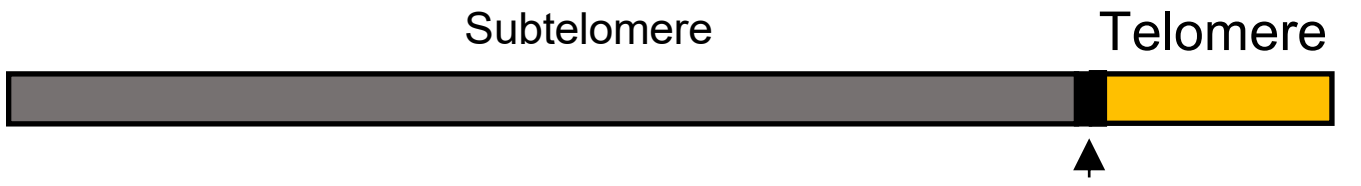


| Position on chromosome arm 1p (nt) | Size | GC (%) | Sequence |
|---|---|---|---|
| 2996-30132; 3025-3042; 3054-3071; 3083-3100; 3112-3129; 3141-3158; 3170-3187; 3199-3216; 3228-3245; 3257-3274; 3286-3303; 3330-3347; 3359-3376; 3429-3446 | 15bp | 77.8 | GGCGCAGGCGCAGAG |

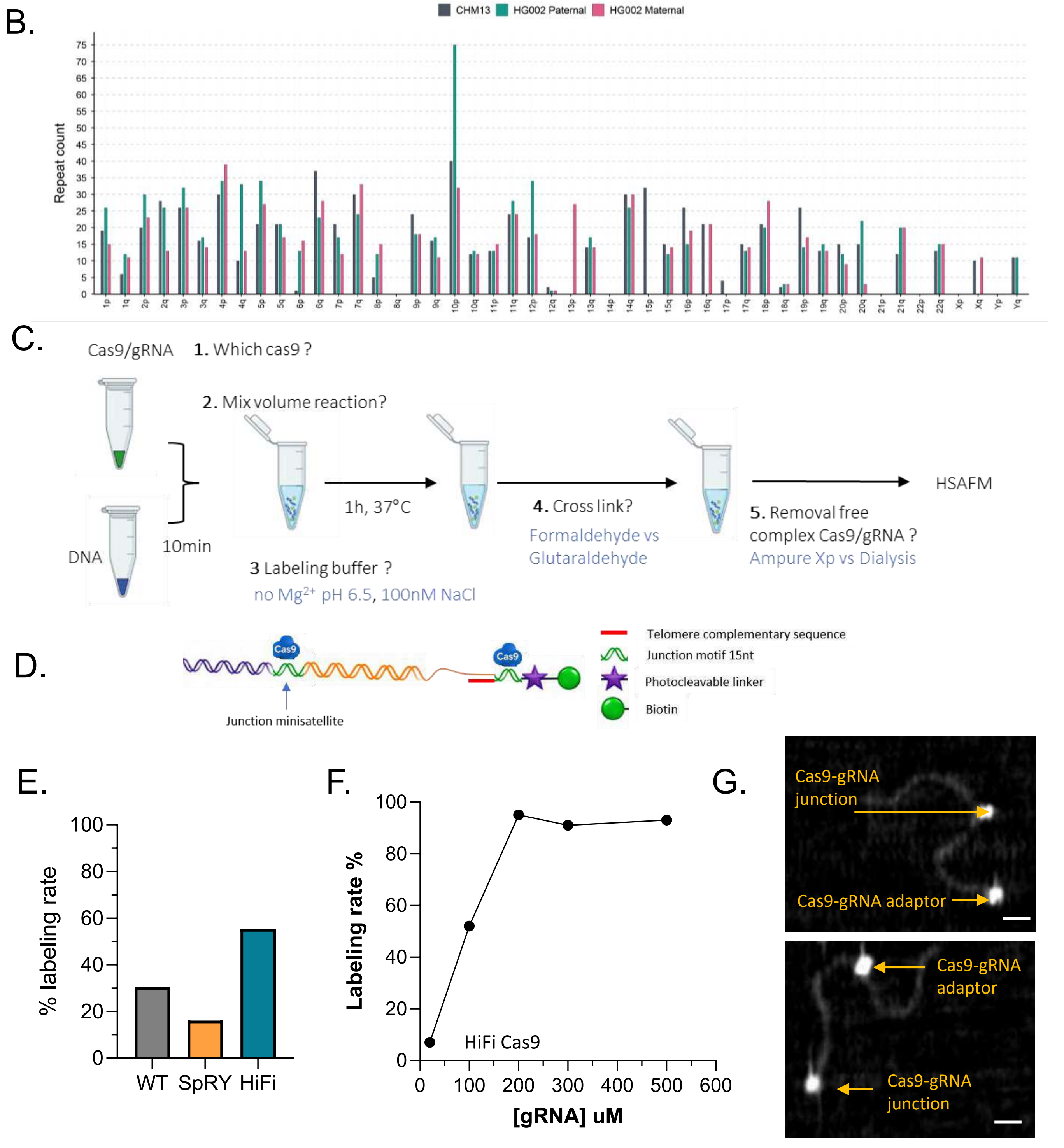


**Supplementary Fig 7. CRISPR–Cas9 labeling of telomere boundaries.** (A) Sequence, genomic positions, and GC content of the recurrent GC-rich minisatellite motif identified at telomere–subtelomere junctions. (B) Frequency of motif occurrence across chromosome ends in the CHM13, HG002 paternal, and HG002 maternal genome assemblies. Motif searches allowed up to two mismatches outside the PAM-proximal region. (C) Workflow for optimization of CRISPR–Cas9 labeling conditions for HS-AFM analysis. (D) Schematic of dual-boundary labeling using Cas9–gRNA complexes targeting the telomere–subtelomere junction motif and the terminal adaptor sequence. (E, F) Comparison of Cas9 variants and gRNA concentrations for boundary labeling. Labeling efficiencies were calculated from analysis of 500 synthetic DNA molecules containing target site for each condition. (G) Representative HS-AFM images showing Cas9–gRNA complexes bound to the telomere–subtelomere junction motif and terminal adaptor sequence.

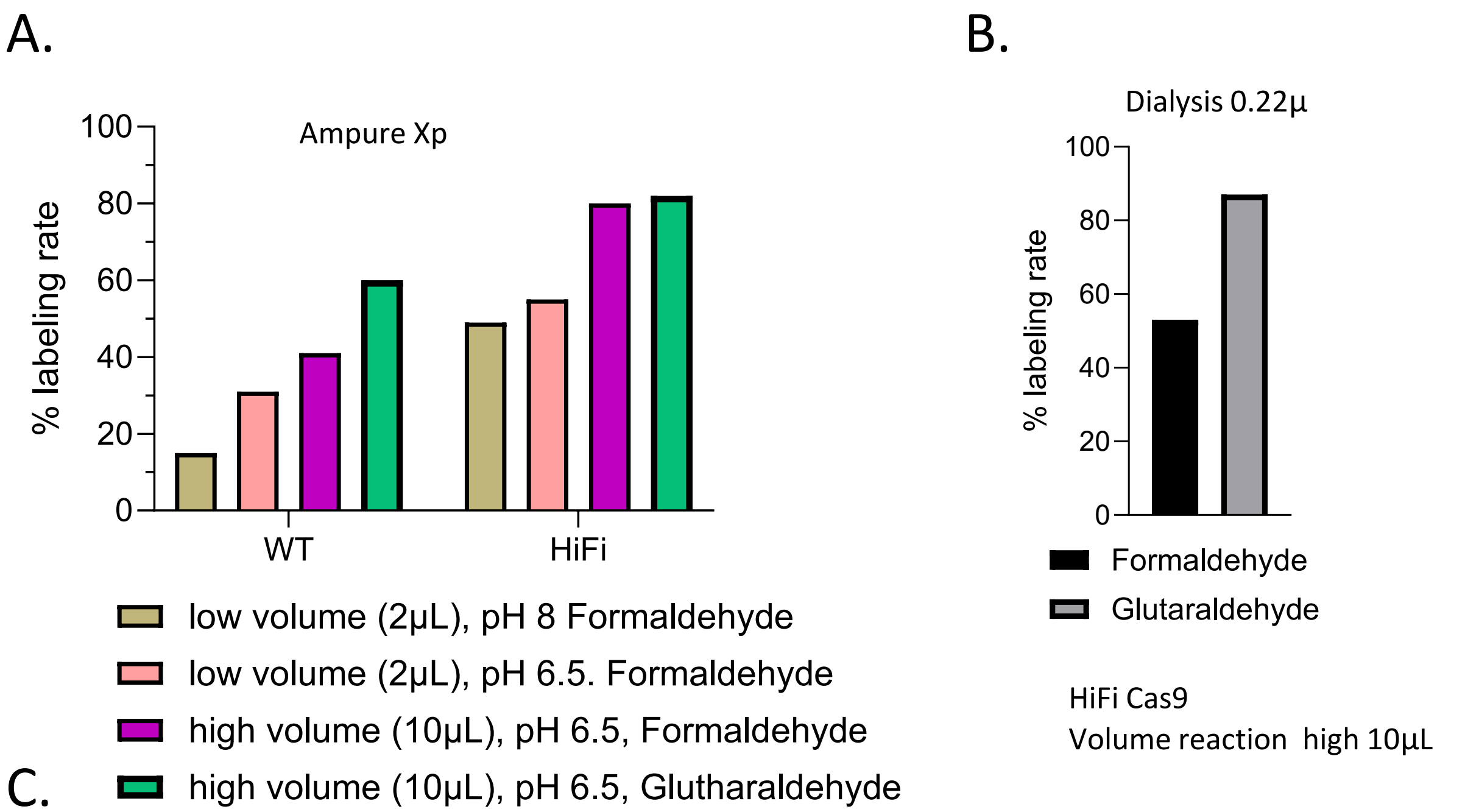


C.

| Conditions | | | Cas9 HiFI/ gRNA labeling | Comments |
|---|---|---|---|---|
| gRNA | Annealing protocol | Concentration at annealing | Labeling rate (%) | |
| Two-parts | One step protocol | 8 µM | 89.3 | |
| Two-parts | Two-steps protocol | 8 µM | 81.4 | |
| Two-parts | Optimized | 8 µM | 93.1 | |
| Two-parts | Optimized | 25 µM | 91.7 | |
| Two-parts | Optimized | 50 µM | 92.5 | |
| Single-part | No annealing | - | 67.4 | Some aggregation |

**Supplementary Fig 8. Optimization of CRISPR–Cas9 boundary labeling conditions.** (A) Comparison of Cas9 labeling efficiencies under different reaction volumes, buffer conditions, and crosslinking reagents following cleanup with AMPure XP beads. WT Cas9 and HiFi Cas9 were evaluated under low-volume (2 µL) and high-volume (10 µL) reaction conditions using formaldehyde or glutaraldehyde fixation. (B) Effect of crosslinking reagent on HiFi Cas9 labeling efficiency following 0.22 µm dialysis cleanup. Labeling efficiencies obtained with formaldehyde and glutaraldehyde fixation are shown. (C) Summary of guide RNA preparation and labeling conditions for HiFi Cas9. Labeling efficiencies were determined from analysis of 500 synthetic DNA molecules containing target site. Different annealing protocols and gRNA concentrations were evaluated to identify conditions maximizing labeling efficiency while minimizing aggregation. The optimized two-part gRNA protocol achieved >90% labeling efficiency.

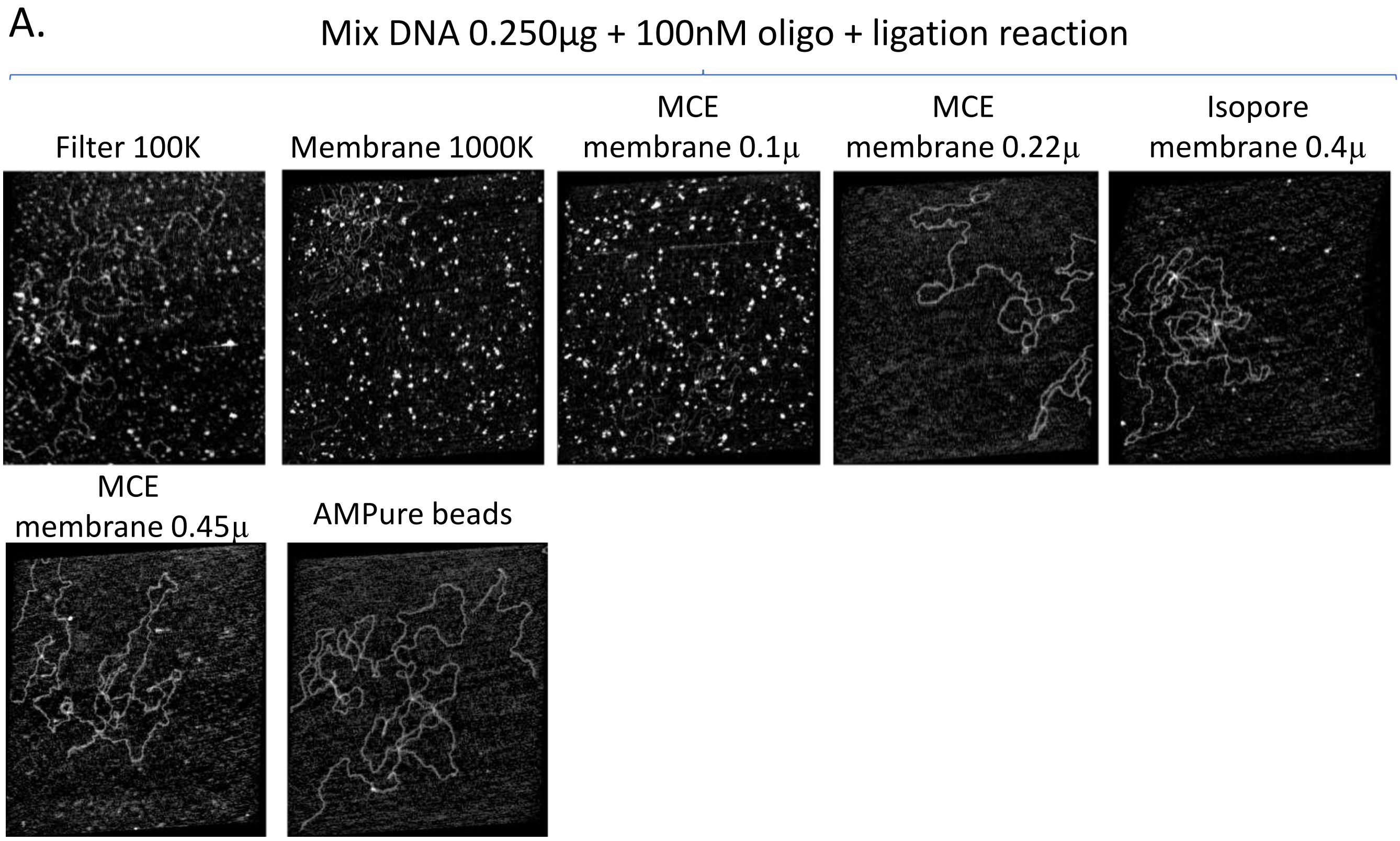


B.

| Beads type | NEB | T1 | M280 | M270 | C1 |
|---|---|---|---|---|---|
| Comment | Size: 1µ<br>Hydrophobic<br>Background associated with beads footprint<br>Good pull down at high DNA concentration (2µg) | Size: 1µ<br>Hydrophobic<br><br>Good pull down at high DNA concentration (2µg) | Size: 2.8µ<br>Hydrophobic<br><br>Good pull down at low DNA concentration (100ng) | Size: 2.8µ<br>Hydrophilic<br><br>High background | Size: 1µ<br>Hydrophilic<br><br>High background |
| Image | 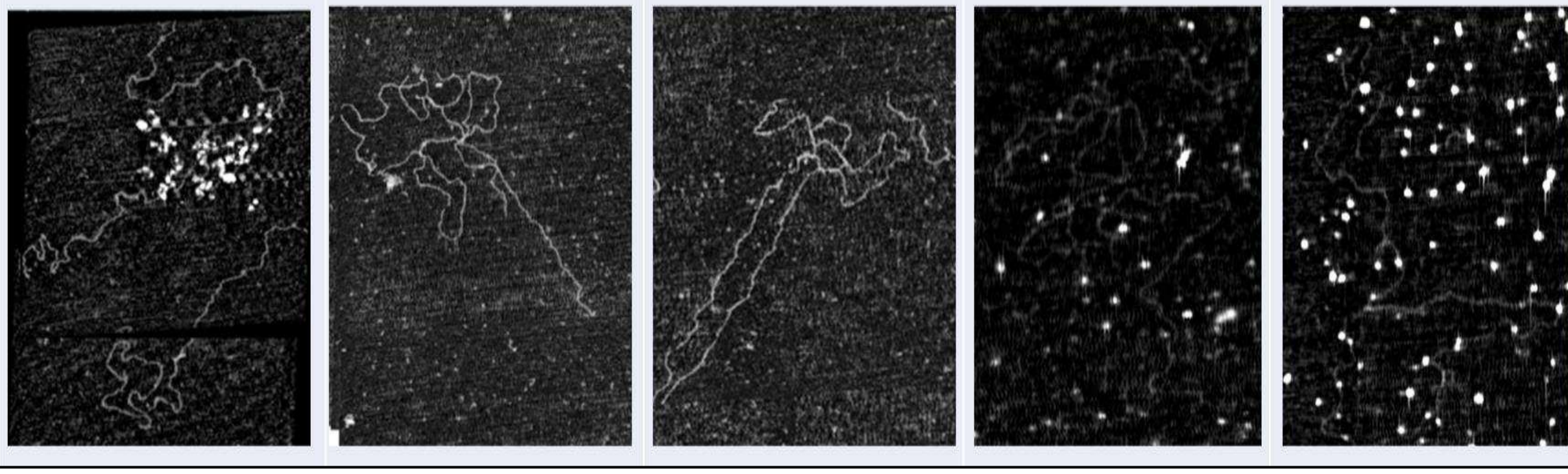 | | | | |

**Supplementary Fig 9. M280 streptavidin magnetic beads and 0.22 µm dialysis provide optimal conditions for HS-AFM analysis.** (A) Representative HS-AFM images of ligation reactions following cleanup with different filtration and purification methods, including 100 kDa filtration, 1000 kDa membrane filtration, 0.1 µm and 0.22 µm MCE membrane filtration, 0.4 µm and 0.45 µm membrane filtration, and AMPure bead purification. The 0.22 µm membrane most effectively removed residual free oligonucleotides while preserving intact DNA molecules. (B) Comparison of streptavidin magnetic bead types for telomere capture and subsequent HS-AFM imaging. Representative images are shown for NEB, T1, M280, M270, and C1 beads. M280 beads provided the best balance between efficient pulldown and low imaging background, whereas M270 and C1 beads generated substantial background signal.

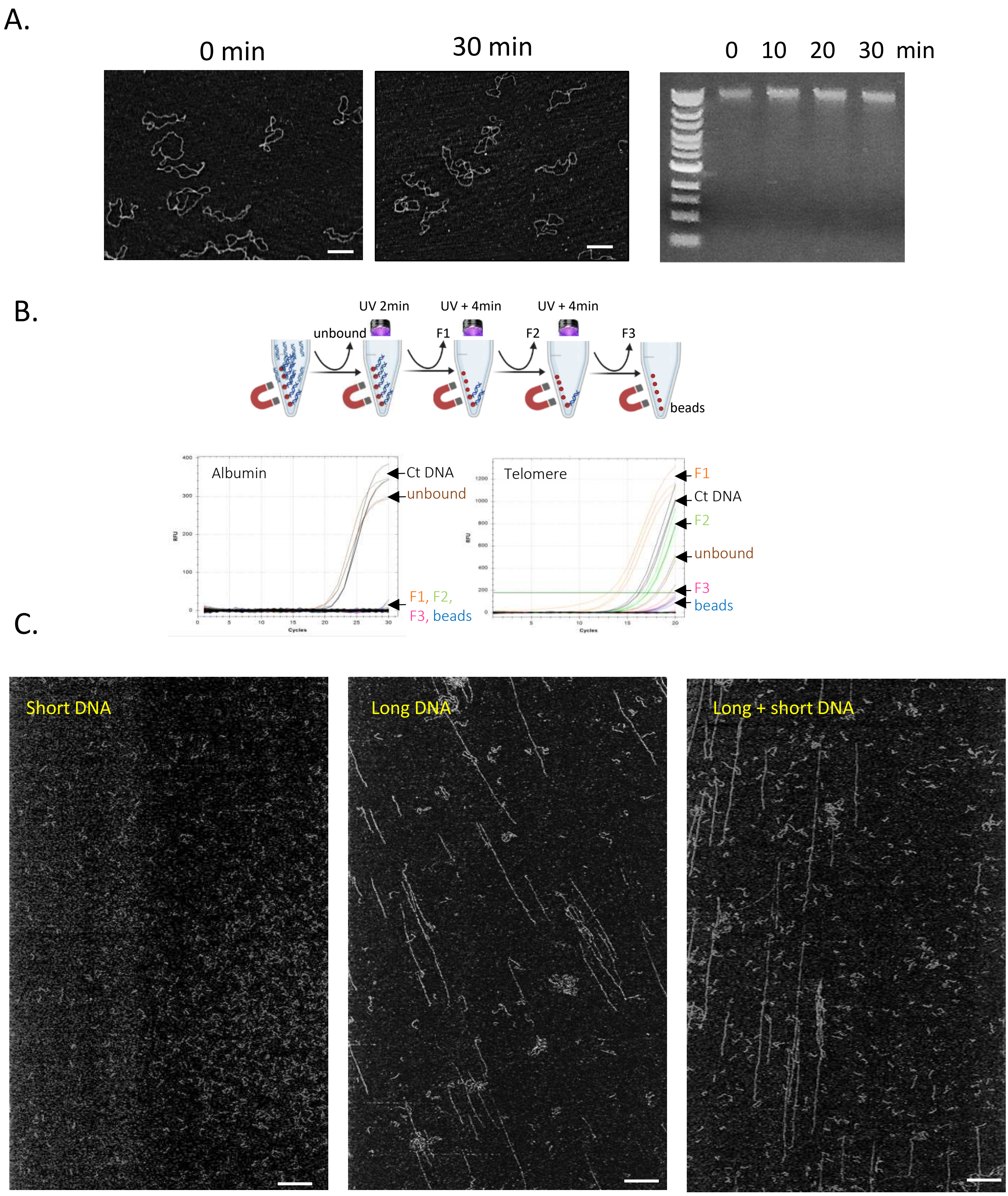


**Supplementary Fig 10. Magnetically directed deposition retains DNA molecules across a broad size range for unbiased telomere size analysis.** (A) Assessment of DNA integrity following exposure to 365-nm UV light for up to 30 min. Representative HS-AFM images (0 and 30 min) and agarose gel electrophoresis (0, 10, 20, and 30 min) showed no detectable DNA fragmentation after UV exposure. (B) Sequential release of telomeric fragments following photocleavage of the linker. Schematic of the release workflow and qPCR analysis of albumin and telomere signals in released fractions demonstrate that the majority of telomeric material was recovered during the initial UV-release steps. (C) Representative HS-AFM images of short DNA, long DNA, and mixed DNA populations following magnetically directed deposition and release onto mica. Comparable recovery of short and long DNA molecules confirms retention across a broad size range without preferential loss of longer molecules.

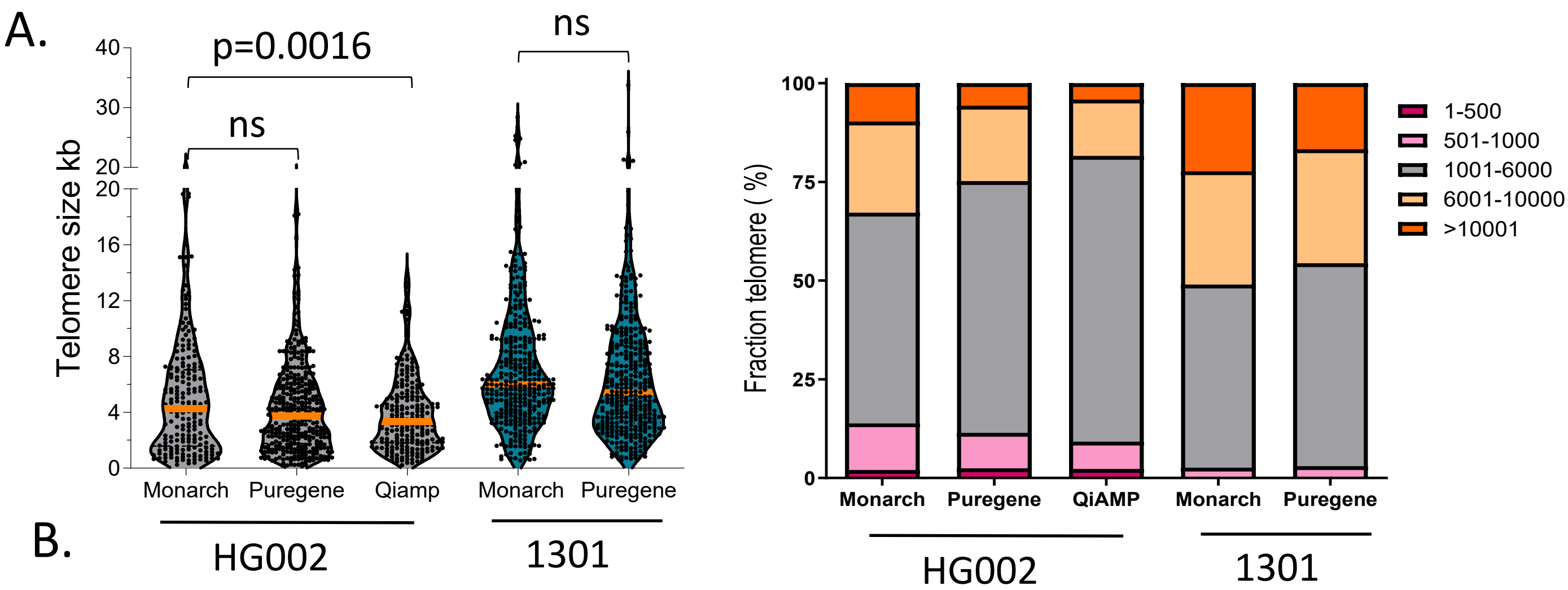


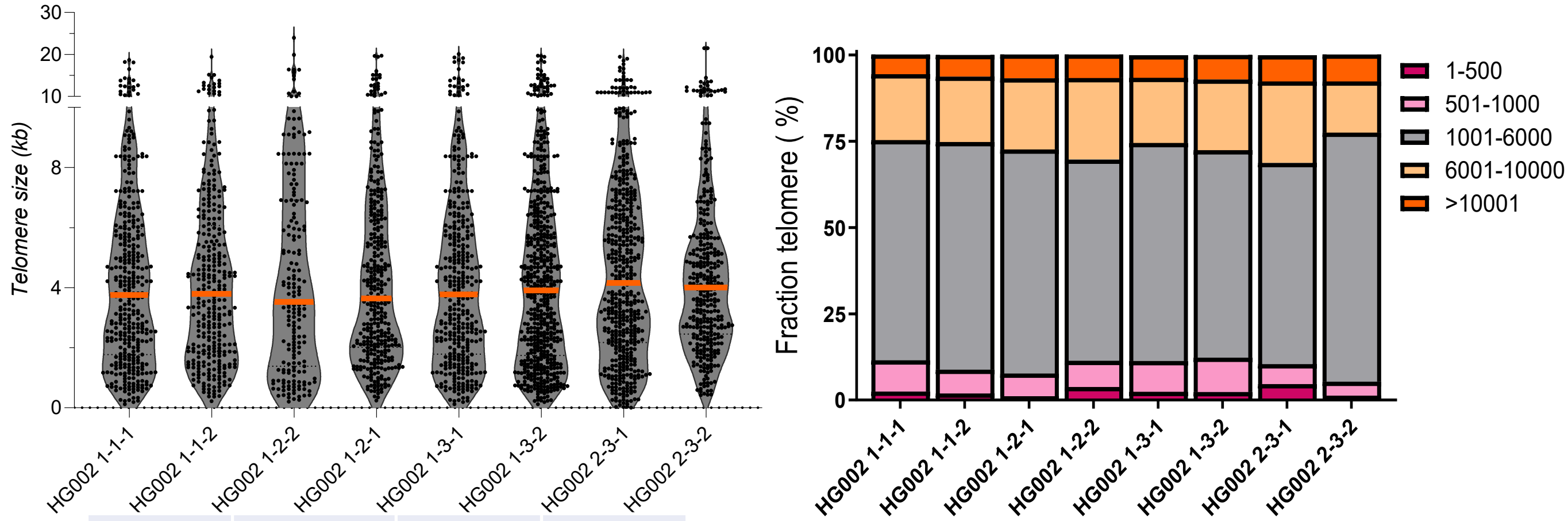


| Cell line | Technician | Extraction | Assay replicate |
|---|---|---|---|
| HG002 | 1 | 1 | 1 |
| HG002 | 1 | 1 | 2 |
| HG002 | 1 | 2 | 1 |
| HG002 | 1 | 2 | 2 |
| HG002 | 1 | 3 | 1 |
| HG002 | 1 | 3 | 2 |
| HG002 | 2 | 3 | 1 |
| HG002 | 2 | 3 | 2 |

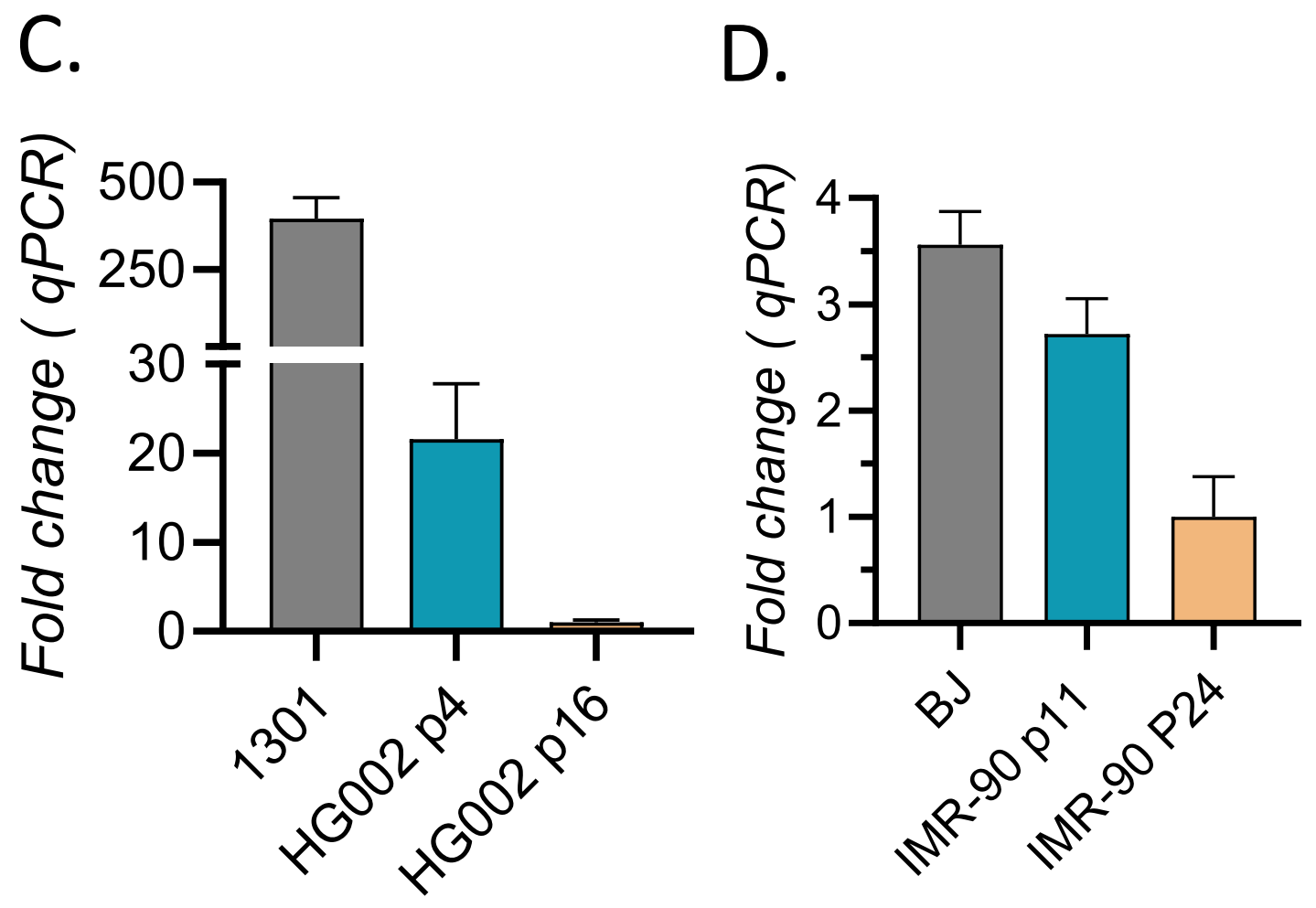


E.

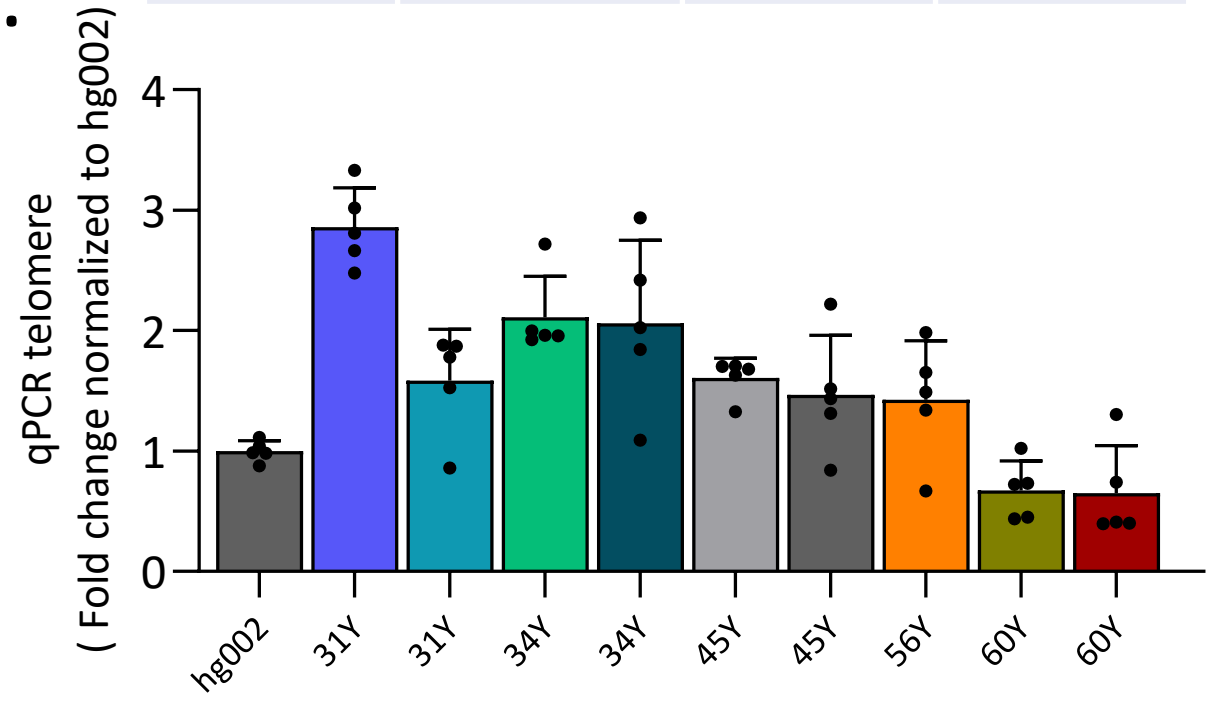


**Supplementary Fig 11.** HS-AFM measurements are robust to DNA extraction yield, operator analysis, and biological variation. (A) Comparison of telomere-length distributions and telomere size categories in HG002 and 1301 cells following DNA extraction with Monarch, Puregene, or QIAamp. Monarch and Puregene produced comparable telomere-length profiles, whereas QIAamp underrepresented longer telomeric fragments. (B) Telomere-length distributions (left) and size-category profiles (right) obtained from independent HG002 DNA extractions and workflow replicates performed by two technicians. No significant differences in telomere-length distributions were detected among extractions, workflow replicates, or technicians. For A and B, each point represents an individual telomere measurement, with approximately 400–500 telomeres analyzed per condition[CJC1.1]. (C) Relative telomere abundance measured by qPCR in reference cell models spanning long-, intermediate-, and short-telomere states. (D) Relative telomere abundance measured by qPCR in BJ and IMR-90 cells at different passages, demonstrating reduced telomere content with culture expansion. (E) Relative telomere abundance measured by qPCR in peripheral blood DNA samples from individuals of different ages, normalized to HG002. Telomere abundance was higher in younger individuals and generally decreased with age. Data are presented as mean ± s.d. from three biological replicates, each assayed in technical triplicate.

## A. CMH13 assembly

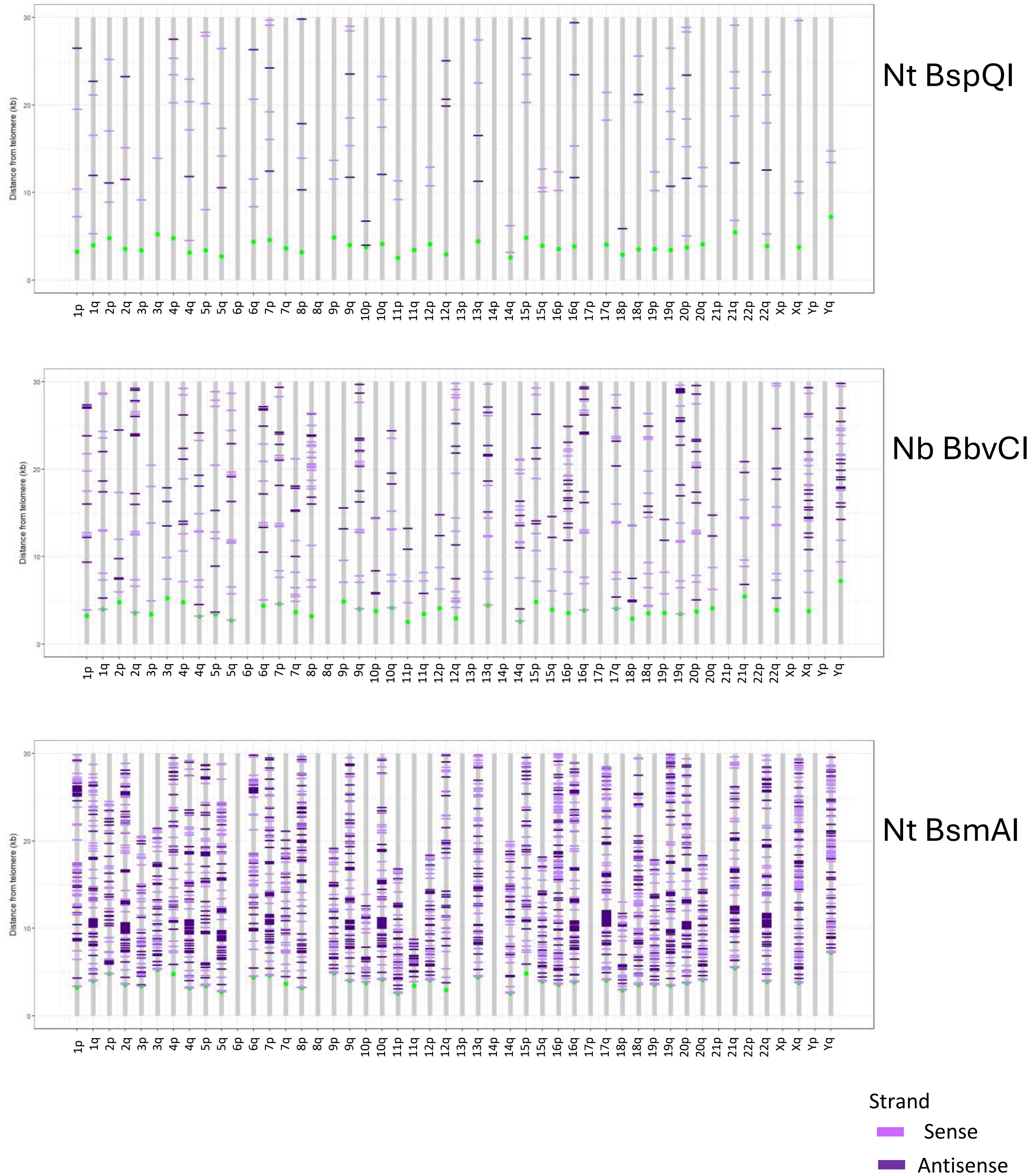

# B. HG002 maternal assembly

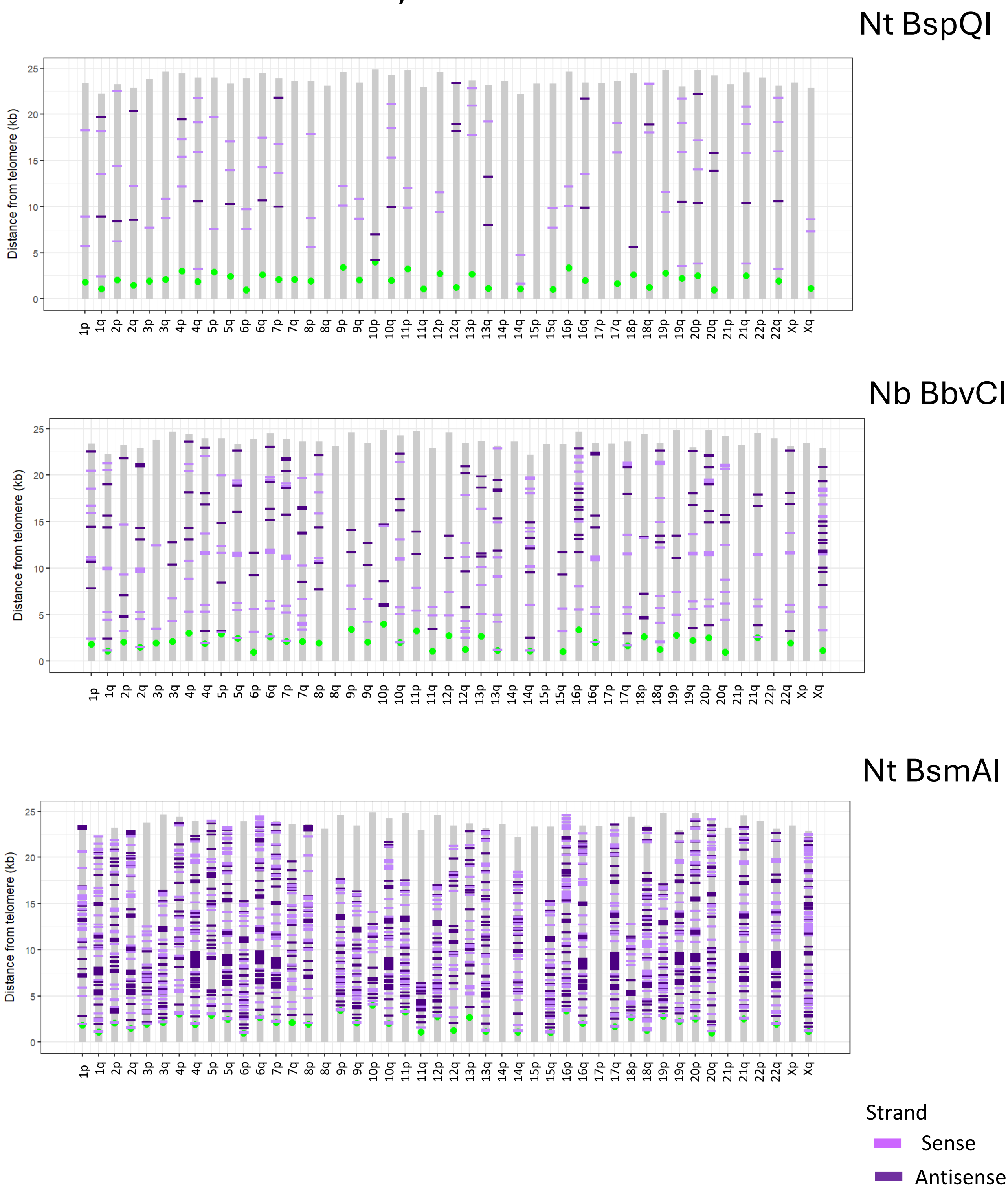

C. HG002 paternal assembly

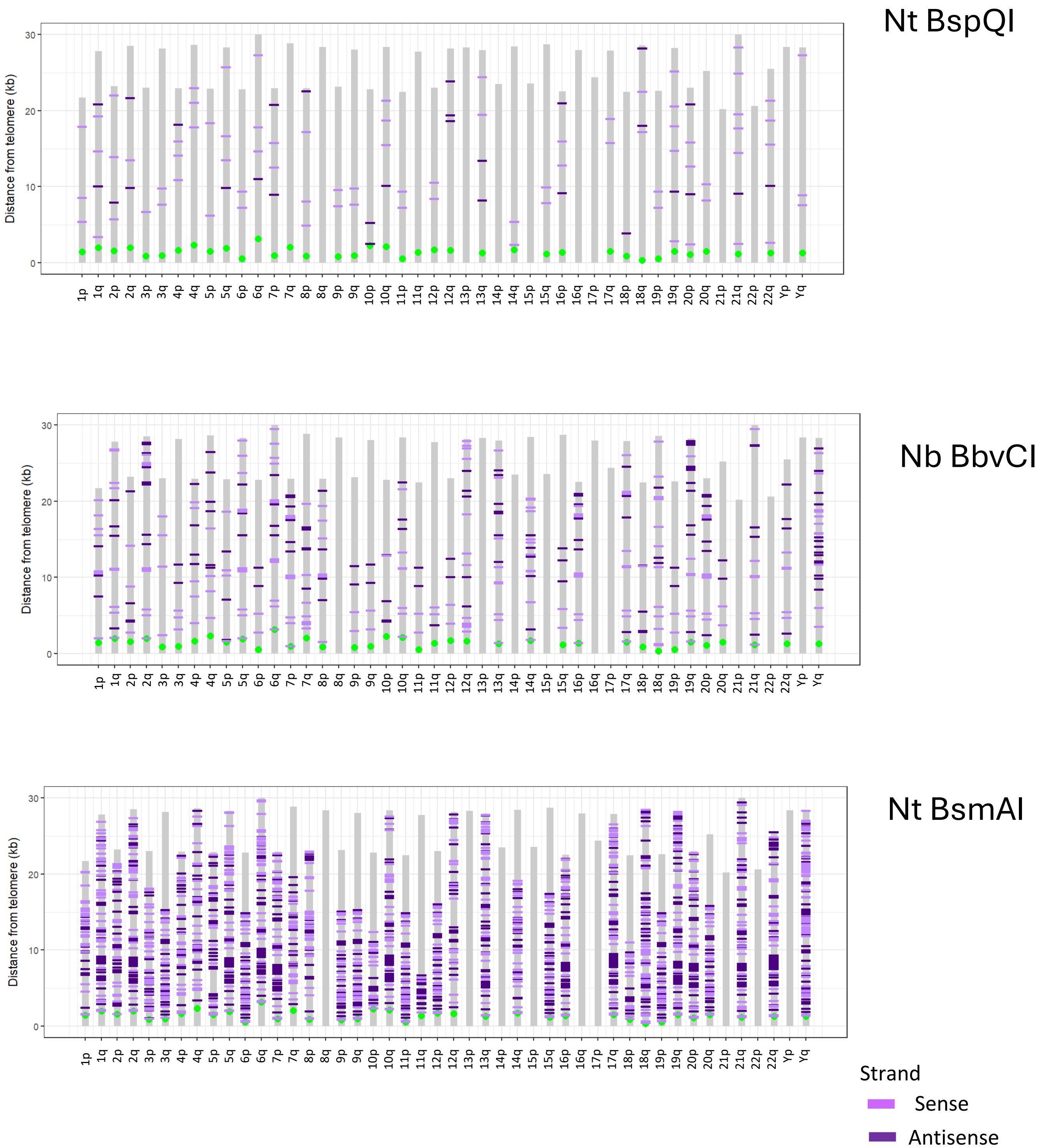


**Supplementary Figure 12 Nt.BspQI produces distinct chromosome-end barcode signatures across human genome assemblies.** (A–C) Predicted Nt.BspQI, Nb.BbvCI, and Nt.BsmAI nicking-site distributions across chromosome ends in the CMH13 (A), HG002 maternal (B), and HG002 paternal (C) assemblies. Chromosome ends are arranged along the x-axis, and distances from the telomere boundary are plotted on the y-axis. Green markers denote telomere boundaries, while purple markers indicate predicted nicking sites, colored according to strand orientation (sense and antisense). Gray vertical bars represent the subtelomeric regions analyzed for barcode generation.

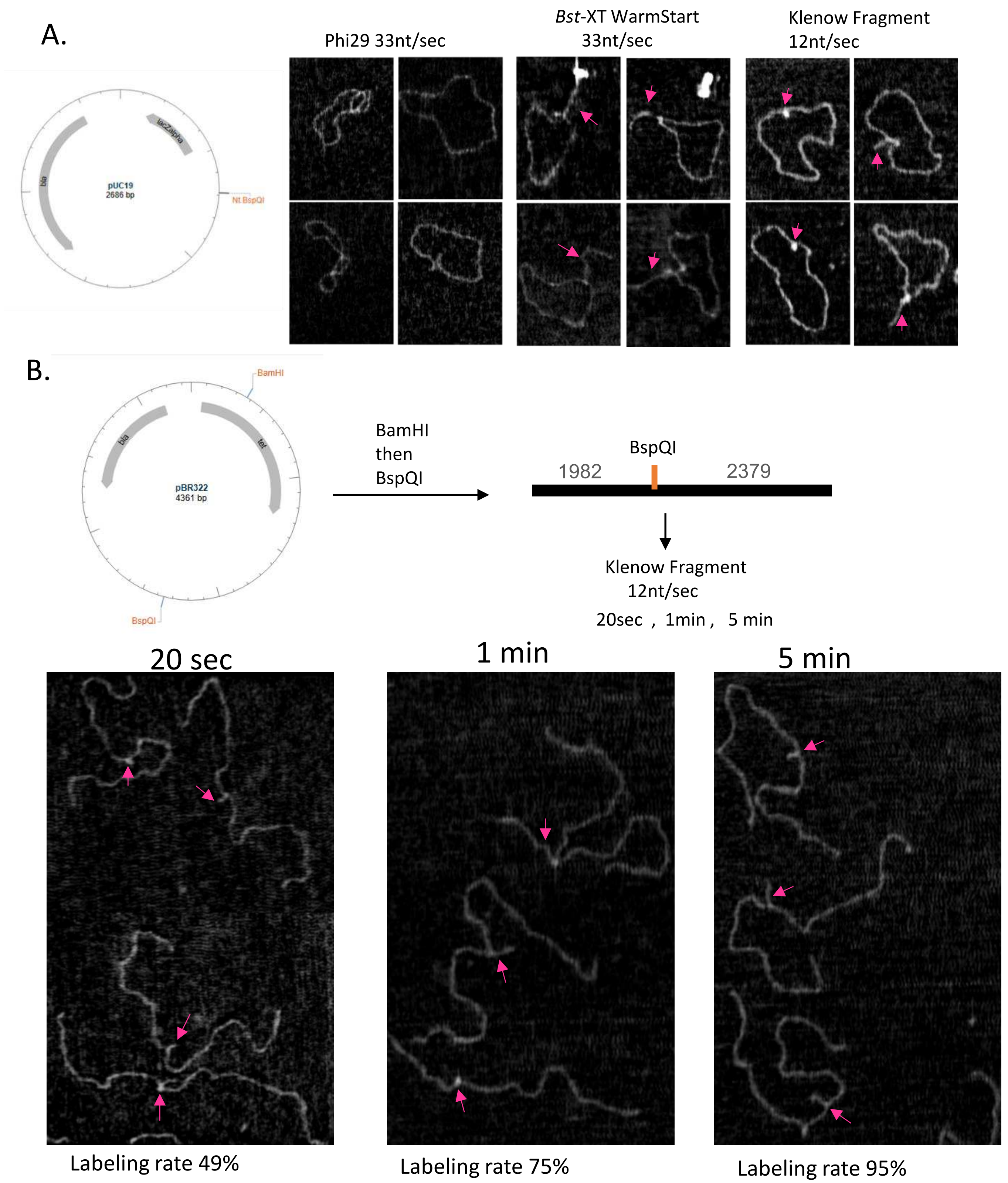


**Supplementary Figure 13. Klenow-mediated strand displacement generates robust DNA-tail labels detectable by HS-AFM.** (A) Evaluation of polymerases for generation of HS-AFM-detectable DNA-tail structures at nicking sites. A pUC19 plasmid containing a single Nt.BstNBI nicking site was subjected to polymerase-mediated strand-displacement synthesis using Phi29 DNA polymerase, Bst-XT WarmStart DNA polymerase, or Klenow Fragment. Representative HS-AFM images are shown for each condition. DNA-tail products generated at the nicking site are indicated by magenta arrows. Phi29 produced few detectable tail structures, whereas Bst-XT WarmStart and Klenow Fragment generated readily visualized DNA tails. Polymerization rates for each enzyme are indicated above the images. (B) Optimization of Klenow-mediated strand-displacement labeling. Linearized pBR322 substrate containing a single Nt.BspQI nicking site at a defined position was generated by BamHI digestion followed by Nt.BspQI nicking. The nicked substrate was subjected to strand-displacement synthesis with Klenow Fragment for 20 s, 1 min, or 5 min. Representative HS-AFM images are shown below, with DNA-tail products indicated by magenta arrows.
